# Screening of tau protein kinase inhibitors in a tauopathy-relevant cell-based model of tau hyperphosphorylation and oligomerization

**DOI:** 10.1371/journal.pone.0224952

**Published:** 2020-07-21

**Authors:** Hamad Yadikar, Isabel Torres, Gabrielle Aiello, Milin Kurup, Zhihui Yang, Fan Lin, Firas Kobeissy, Richard Yost, Kevin K. Wang

**Affiliations:** 1 Program for Neurotrauma, Neuroproteomics & Biomarkers Research, Departments of Emergency Medicine, Psychiatry, Neuroscience and Chemistry, University of Florida, Gainesville, Florida, United States of America; 2 Department of Biological Sciences, Faculty of Science, Kuwait University, Safat, Kuwait; 3 Department of Chemistry, Chemistry Laboratory Building, University of Florida, Gainesville, FL, United States of America; 4 Faculty of Medicine, American University of Beirut Medical Center, Beirut, Lebanon; 5 Brain Rehabilitation Research Center, Malcom Randall VA Medical Center, Gainesville, FL, United States of America; McGill University, CANADA

## Abstract

Tauopathies are a class of neurodegenerative disorders characterized by abnormal deposition of post-translationally modified tau protein in the human brain. Tauopathies are associated with Alzheimer’s disease (AD), chronic traumatic encephalopathy (CTE), and other diseases. Hyperphosphorylation increases tau tendency to aggregate and form neurofibrillary tangles (NFT), a pathological hallmark of AD. In this study, okadaic acid (OA, 100 nM), a protein phosphatase 1/2A inhibitor, was treated for 24h in mouse neuroblastoma (N2a) and differentiated rat primary neuronal cortical cell cultures (CTX) to induce tau-hyperphosphorylation and oligomerization as a cell-based tauopathy model. Following the treatments, the effectiveness of different kinase inhibitors was assessed using the tauopathy-relevant tau antibodies through tau-immunoblotting, including the sites: pSer202/pThr205 (AT8), pThr181 (AT270), pSer202 (CP13), pSer396/pSer404 (PHF-1), and pThr231 (RZ3). OA-treated samples induced tau phosphorylation and oligomerization at all tested epitopes, forming a monomeric band (46–67 kDa) and oligomeric bands (170 kDa and 240 kDa). We found that TBB (a casein kinase II inhibitor), AR and LiCl (GSK-3 inhibitors), cyclosporin A (calcineurin inhibitor), and Saracatinib (Fyn kinase inhibitor) caused robust inhibition of OA-induced monomeric and oligomeric p-tau in both N2a and CTX culture. Additionally, a cyclin-dependent kinase 5 inhibitor (Roscovitine) and a calcium chelator (EGTA) showed contrasting results between the two neuronal cultures. This study provides a comprehensive view of potential drug candidates (TBB, CsA, AR, and Saracatinib), and their efficacy against tau hyperphosphorylation and oligomerization processes. These findings warrant further experimentation, possibly including animal models of tauopathies, which may provide a putative Neurotherapy for AD, CTE, and other forms of tauopathy-induced neurodegenerative diseases.

## Introduction

Tauopathy is a class of neurodegenerative conditions that are associated with pathological phosphorylated tau protein accumulation in the human brain. Tauopathy has been associated with several clinicopathological conditions, including chronic traumatic encephalopathy (CTE) [1], traumatic brain injuries [2], post-traumatic stress disorder [3], and Alzheimer’s disease (AD) [4, 5]. Tau is a structural protein whose function is to promote microtubule stabilization and assembly, which are controlled by its phosphorylation state [6–8]. In humans, the tau gene encodes the tau protein and is located on chromosome 17q21 [9]. The main tau protein is encoded by 11 exons, which are subjected to alternative splicing of exon two, three, and ten forming six isoforms. The six tau isoforms range from 352 to 441 amino acids. Tau isoforms vary in either having zero, one, or two N-terminal inserts (exons two and three) and three or four repeats region at the C-terminal region (exon 10) [10, 11]. SDS-PAGE gel experiments and immunohistochemical analyses showed that tau produced at least six bands with distinct molecular masses of 48 kDa–67kDa and a hallmark spacing sequence [12, 13]. The brain displayed tau at significantly larger amounts than other regions, with the tau-441 isoform being prevalent in two-month-old adult mice [13].

Tau can be subjected to a sequence of post-translational modifications that may attenuate the protein’s structure, function, turnover, or can lead to multimeric aggregation. In addition to phosphorylation, these modifications include acetylation [14], methylation [15, 16], nitration [17], glycosylation [18], sumoylation [19]. Post-translational phosphorylation is still the most well-studied alteration of tau since it is considered a hallmark across all tauopathies. Tau protein consists of 79 potential phosphorylatable serine and threonine sites on the longest isoform. At least thirty tau phosphorylation sites have been reported in healthy conditions. Tau’s phosphorylation state and its ability to interact with microtubule proteins are regulated by various protein kinases and phosphatases [20, 21]. Imbalances in the activities of tau kinases and phosphatases can cause tau to become phosphorylated at specific residues leading to a higher tendency to dissociate from microtubules. Abnormally dissociated tau have a higher susceptibility of forming larger protein aggregates, filament assembly, and bundling of paired helical filaments (PHF) into neurofibrillary tangles (NFT) leading to cellular neurotoxicity [6–8, 22, 23].

The substantial link between tau phosphorylation and disease has established the foundation for the discovery of potentially restorative tau kinase inhibitors. Tau phosphorylation is carried out by a host of different kinases under physiological conditions. Abnormal activities of tau kinases have been associated with AD, including kinases such as Src family kinase, Ca^2+^/calmodulin-dependent protein kinase II (CaMKII); cyclin-dependent kinase 5 (CDK5); casein kinase (I/II); dual-specificity tyrosine kinase phosphorylation and regulated kinase-1A/2 (DYRK1A/2), glycogen synthase-3 (GSK-3), and tyrosine kinase Fyn [24, 25]. Notably, a study reported that the hippocampus and temporal cortex regions throughout the brain have high levels of CKII in AD when compared to controls [26]. The tyrosine kinase Fyn has been highly researched for its implications with tau and neurodegeneration in the post-synaptic N-methyl-D-aspartate receptors (NMDAR) [24, 27–29]. Fyn phosphorylate tau in the N-terminal domain in neurons and plays a fundamental role in the amyloid signal transduction [24]. A small-molecule Fyn inhibitor, Saracatinib (AZD0530), demonstrated memory deficits reduction in transgenic mice [24].

Several approaches to treat tauopathic conditions have been investigated, including targeting tau kinases [30], activation of tau phosphatases [31], enhancing microtubule stabilization [32], tau immunotherapy [33], tau clearance [2], tau aggregation inhibition [34]. Since *in vivo* tau-hyperphosphorylation results from multiple kinase activities, a single effective strategy to reverse tauopathies is still an open question. The inhibition of tau kinases using pharmaceutical drugs can lead to decreased levels of the hyperphosphorylated tau protein, thereby less aggregated tau [35–40]. Several tau kinase inhibitors are in clinical trials to treat tauopathies-related diseases [41]. Besides, over the last few years, the production of protein kinase inhibitors has continued to provide an environment of intensive preclinical activity, given the difficulties that these strategies face concerning toxicity and selectivity. The most progressive protein kinase inhibition approach in the clinic thus far has been targeted at GSK-3β protein [38, 42]. However, LiCl is not specific for GSK-3β against GSK-3α and may have some off-target consequences, which render it difficult to determine its capacity to inhibit tau phosphorylation through GSK-3β. A selective GSK3 inhibitor AR-A014418 has also been shown to suppress tau phosphorylation, aggregated tau, and axonal deterioration after ingestion for one month in tau transgenic mice [43]. A clinical study was initiated for AR-A014418 analog, but progress has been halted since then.

Tideglusib, a non-ATP active GSK-3β antagonist, reduced tau phosphorylation, diminished Aβ accumulation, the proliferation of astrocytes, and cognitive defects in an *in vivo* AD model [44]. While GSK-3 is a largely conserved kinase, only a few GSK-3 antagonists (synthetic or natural) studied in the preclinical stage have entered clinical trials. Given many promising findings in animal models, the reality that GSK-3β is omnipresent and biologically active in many cellular mechanisms has posed numerous questions and major toxicity obstacles for preclinical and therapeutic long-term studies [45, 46]. The initial clinical trial phase I drug, as a GSK-3ß antagonist, was AZD2558 [47]. This remarkably specific GSK-3β antagonist effectively decreased tau phosphorylation and *in vivo* gliosis. The extent of nonspecific target outcomes correlated with *in vivo* delivery prohibited this medication from being evaluated for AD. A further effort was generated with AZD1080, previously shown to suppress tau phosphorylation *in vitro* and in preclinical studies [47]. This drug prolonged delivery was also associated with major adverse effects, and stage I analysis was halted [47]. Until today, only GSK-3 antagonist Tideglusib has completed stage II clinical trials to combat AD and supranuclear palsy [38, 48, 49]. Tideglusib has been evaluated in two limited clinical phases II studies in adults with mild to severe AD, demonstrating reasonable safety and effectiveness and yet no major clinical improvements [46]. It has been shown in AD and various other tauopathies that, tau is abnormally phosphorylated at Ser202, Ser396/404, Thr181, Thr205, and Thr231 [50, 51]. The phosphorylation profile of tau residues at Ser202/Thr205 has been well-characterized in AD cases based on using specific antibodies [52]. Analyzing these phosphorylation sites helps to show a pattern of relationships between tau protein phosphorylation and pathology. Protein phosphatase stimulation has also been proposed as an alternative strategy for reducing tau phosphorylation, especially in the context of the vital brain tau phosphatase (PP2A) [53]. However, the triggering of PP2A is difficult, because it has a broad substrate specificity and multiple regulatory subunits, rendering it challenging for the appropriate group to be aimed at in the correct position and time [54]. Nonspecific kinase inhibitors were proposed, hoping that several kinases phosphorylate tau, and reaching a few of these will partly minimize tau phosphorylation, thus reducing harmful effects. One of these drugs, SRN-003-556, which suppresses GSK3, CDK1, protein kinase A (PKA), protein kinase C (PKC), and mitogen-activated protein kinase 1 (MAPK1) to similar levels lowered tau phosphorylation and neuronal impairment in tau transgenic mouse model without influencing NFT [55].

Okadaic acid (OA), a protein phosphatase 1 and 2A (PP1/PP2A) inhibitor, induces tau hyperphosphorylation at pathological sites in both animal and cell-based models [56–58]. OA inhibition of tau phosphatases allows the activation of multiple tau kinases, leading to its hyperphosphorylation [59, 60]. It has been shown that OA treatment in wild-type mice causes tauopathy-related abnormality in different regions of the brain [61]. In this study, mouse neuroblastoma culture (N2a) and rat primary cerebrocortical neuronal (CTX) culture were treated with OA, to induce tau hyperphosphorylation and oligomerization mimicking a tauopathy-relevant condition. In these experiments, we used the OA-induced tauopathy culture model to screen for different tau kinase inhibitors implementing immunoblotting and phospho-specific tau antibodies. Thus, it was hypothesized that using OA-induced tau hyperphosphorylation and aggregation as a tauopathy model to screen for kinase inhibitors would translate into putative neurotherapeutic targets for tauopathies-related disorders. Data from this work has shown that the different treatments inhibited OA-induced tau hyperphosphorylation and oligomerization. This side-by-side overview both highlights targets not well described, and corroborates with data from targets previously studied, to be assessed in different relevant tauopathy-related *in vivo* models.

## Materials and methods

### Phosphorylation inhibitors

Ethylene glycol-bis(β-aminoethyl ether)-N,N,N',N'-tetraacetic acid (**EGTA**) (Sigma-Aldrich, St-Louis, MO, USA), Dithiothreitol (**DTT**) (Sigma-Aldrich), Lithium chloride (**LiCl**) (Sigma-Aldrich), N-(4-methoxybenzyl)-N'-(5-nitro-1,3-thiazol-2-yl)urea (**AR-A014418**) (Sigma-Aldrich), (9S,10R,12R)-2,3,9,10,11,12-Hexahydro-10-hydroxy-9-methyl-1-oxo-9,12-epoxy-1H-diindolo[1,2,3-fg:3',2',1'-kl]pyrrolo[3,4-i][1,6]benzodiazocine-10-carboxylic acid methyl ester (**K252a**) (Sigma-Aldrich), (2R)-2-1-butanol (**Roscovitine**) (Sigma-Aldrich), 4,5,6,7-Tetrabromo-2-azabenzimidazole (**TBB**) (Sigma-Aldrich), 1-(7-methoxyquinolin-4-yl)-3-(6-(trifluoromethyl)pyridin-2-yl)urea (**A-1070722**) (Sigma-Aldrich), cyclosporine A (Sigma-Aldrich), N-(5-chloro-1,3-benzodioxol-4-yl)-7-[2-(4-methylpiperazin-1-yl)ethoxy]-5-(tetrahydro-2H-pyran-4-yloxy)quinazolin-4-amine (**Saracatinib**) (Selleck Chemicals, Houston TX), (5S,6R,7R,9R)-6-methoxy-5-methyl-7-(methylamino)-6,7,8,9,15,16-hexahydro-17-oxa-4b,9a,15-triaza-5,9-methanodibenzo[b,h]cyclonona[jkl]cyclopenta[e]-as-indacen-14(5h)-one (**STS**) (ab120056; Abcam, Cambridge, MA, USA), Z-Asp-2,6-Dichlorobenzoyloxymethyl Ketone (**Z-DCB**) (Cayman Chemical, Ann Arbor Michigan) and okadaic acid (Cell Signaling Technology, Danvers, MA). SNJ-1945 was a gift from (Senju Pharmaceutical Co. Ltd., Kobe, Japan) **(Table 1)**.

**Table 1:** Phosphatase, kinase inhibitor and other pharmacological agents used in the study.

Agent	Full Name / Function	Target	Affinity in vitro (K_i_)	Cross- reactivities	IC_50_ (cell-based assay)
**Phosphatase Inhibitors**					
**OA**	Okadaic Acid, Serine/Threonine phosphatase activity	PP1, PP2A inhibitor	150 nM, 32 pM [62]	----------------	0.1 μM [63]
**CsA**	Cyclosporin A/calcium dependent protein phosphatase—immunosuppressant	Calcineurin (PP3)	0.98 μM [64]	FK-506	55 μM [65]
**Kinase Inhibitors**					
**LiCl**	Lithium Chloride–acts by competing for magnesium.	GSK3β	1–2 mM [66]	----------------	1–2 mM [67]
**AR-A014418**	N-(4-methoxybenzyl)-N'-(5-nitro-1,3-thiazol-2-yl) urea, antidepressant. Inhibits in an ATP competitive manner	GSK3β	38 nM [68]	----------------	104±27 nM [68]
**A-1070722**	1-(7-methoxyquinolin-4-yl)-3-(6-(trifluoromethyl) pyridin-2-yl) urea Brain penetrant. Inhibits in an ATP competitive manner.	GSK-3α and GSK-3β	0.6 nM [69]	----------------	35–66 nM [70]
**K252a**	Staurosporine analog, non-selective cell-permeable Protein Kinase Inhibitor	PKA, PKC, PKG, CaMK, and phosphorylase kinase, and others	1.8–20 nM [71]	Broad kinase inhibitor	1.3–3 μM [72]
**STS**	Staurosporine, highly non-selective cell-permeable Protein Kinase Inhibitor	Pan Protein Kinase Inhibitor	3–15 nM	Broad kinase inhibitor	0.5 μM [73]
**AZD0530**	Saracatinib, anti-invasive and anti-tumor activities	Src/Fyn Tyrosine Kinase inhibitor	5–10 nM [29]	Brc-Abl tyrosine kinase	1–10 μM [74]
**Roscovitine**	Seliciclib, competes for the ATP binding sites, apoptotic and antineoplastic activity	CDK5/P35 inhibitor	0.2 μM [75]	Pyridoxal Kinase (non-protein target)	10 μM [76]
**TBB**	4,5,6,7-tetrabromobenzotriazole, Acts in an ATP/GTP-competitive manner by binding to the Val66 residue of casein kinase-2.	CKII inhibitor	80–210 nM [77]	----------------	10 μM (from the present study)
**Calcium Chelators**					
**EGTA**	ethylene glycol-bis (β-aminoethyl ether)-N, N, N’, N'-tetraacetic acid, chelator of divalent cations.	divalent ion chelator (Ca^2+^ /Mg^2+^)	10 nM [78]	Tyr kinase (500 nM)	2–5 mM [78]
**Other Inhibitors**					
**SNJ-1945**	Amphipathic ketoamide–neuroprotective cell-permeable calpain inhibitor	Calpain 1, 2 inhibitors	100 nM [79]	----------------	20–30 μM [79]
**Z-DCB**	Z-Asp-2,6-Dichlorobenzoyloxymethyl Ketone, Inactivates the interleukin-1β-converting enzyme	Pan-Caspase inhibitor	1–10 μM [80]	Inhibit the production of cytokines in human peripheral blood mononuclear cells and T cell proliferation	20–50 μM [81]

### Antibodies

The following commercial antibodies that were used in this study include (with reference to the validation analysis): AT8 (mouse monoclonal, pSer202/pThr205, 1/1000; Fisher cat#MN1060; Lot number PI205175) [82, 83], AT270 (mouse monoclonal, pThr181, 1/1000; Fisher cat#MN1050; Lot number PK208457) [84], total tau DAKO (rabbit polyclonal, cat#A0024, 1/5000, CiteAb, England) [85], anti-αII-spectrin (mouse monoclonal, cat# BML-FG6090-0100, ENZO Life Sciences, Farmingdale, NY, USA, 1/5000), β-actin (rabbit polyclonal, cat# ab8227, abcam, Cambridge, MA, USA, 1/3000). Anti-mouse and anti-rabbit IgG (whole molecule)−Alkaline Phosphatase antibody were produced in rabbit (polyclonal, Cat#A1902-1ML; Merek, Darmsstadt, Germany). The non-commercial tau antibodies were total tau mouse monoclonal antibodies: DA9 (a.a. 102–140, 1/1000) [86], DA31 (aa150-190, 1/1000) [86], PHF-1 (mouse monoclonal, pSer396/pSer404, 1/1000) [83], CP13 (mouse monoclonal, pSer202, 1/1000) [87], and RZ3 (mouse monoclonal, pThr231, 1/1000) [88]. (these antibodies were a generous gift from Dr. Peter Davies (Feistein Institute for Medical Research, Manhasset, NY, USA)**(Table 2)**.

**Table 2:** Antibodies used in this study.

Clone name	Epitope*	Supplier (Catalog#)	MAb/ PAb
**Phospho Tau antibodies**			
AT8	pSer202/pThr205	Fisher-Thermo (MN1020)	Mouse MAb
AT270	pThr181	Fisher-Thermo (MN1050)	Mouse MAb
RZ3	pThr231	Peter Davies, Albert Einstein College of Medicine, Bronx, NY	Mouse Mab
CP13	pSer202	Peter Davies, Albert Einstein College of Medicine, Bronx, NY	Mouse MAb
PHF-1	pSer396/pSer404	Peter Davies, Albert Einstein College of Medicine, Bronx, NY	Mouse Mab
**Total Tau Antibodies**			
DA9	aa102-140	Peter Davies, Albert Einstein College of Medicine, Bronx, NY	Mouse Mab
DA31	aa150–190	Peter Davies, Albert Einstein College of Medicine, Bronx, NY	Mouse Mab

*Epitope based on human Tau-441 sequence.

### Cell lines and media

Mouse neuroblastoma N2a cell culture were purchased directly from American Type Culture Collection company (ATCC^®^ CCL-131™, Manassas, VA, USA) and were grown as recommended by the manufacturer [89]. Endogenous tau was used in all experiments. The cells were grown at 1:1 Dulbecco’s modified Eagle medium: reduced serum Eagle’s minimum essential media (DMEM: Opti-MEM) supplemented with 5% FBS (Thermo-Fisher), 100 units/ml penicillin and 0.1 mg/ml streptomycin. Cells were incubated at 37^º^C in a humidified 5% CO_2_-containing atmosphere.

### Primary cerebrocortical neuronal cultures

Established rat primary cerebrocortical neuronal culture (Thermofisher; Cat. No. A10840) harvested from a homogenized pool of day ten Sprague–Dawley rat brains and plated on poly-L-lysine-coated (0.01% (w/v)) 12-well culture plates (Erie Scientific, Portsmouth, NH, USA), similar to previously described methods [90] at a density of 4.36 × 10^5^ cells/ml. Cultures were grown in Neurobasal® media (Thermo Fisher), supplemented with 1% B-27 (Thermo Fisher), one mM Glutamine (Thermo Fisher), and incubated at 37°C in a humidified 5% CO_2_-containing atmosphere. The medium was replaced every three days.

### Cell treatments

In all experimental conditions, endogenous tau was used to asses the tau phosphorylation levels. For N2a cell culture treatments, complete media were replaced with serum-free DMEM media. For CTX primary cultures, all experiments were performed after ten days in culture, and the media was replaced with Neurobasal® media supplemented with 0.5% B-27. For both CTX and N2a culture, SNJ-1945 (S, 100 μM) and Z-DCB (Z, 60 μM) were added to all experimental conditions before the treatment for 1h. Next, okadaic acid (OA; 100 nM) was added for 24h followed by protein kinase inhibitors for 6h. The protein kinase inhibitors used included: K252a (10 μM), AR-A014418 (60 μM), A-1070722 (60 μM), Saracatinib (100 μM), LiCl (5 mM) TBB (30 μM), EGTA (five mM), Roscovitine (60 μM), STS (0.5 μM), CsA (60 μM) (if added) (**Table 1**).

### Cell lysate collection and preparation

The culture lysate harvesting of N2a cells and CTX culture were identical. After the treatment, conditioned media were collected from each well and added into separate tubes on ice and centrifuged at 10,000 x g for 10 min at 4°C. Lysis buffer was added to the attached cells on the 12-well plates (100 μl per well). The Triton-X lysis buffer included: 1mM DTT, 1% phosphatase inhibitors (Sigma), 1% Mini-Complete protease inhibitor cocktail tablet (Roche Biochemicals), and 1% Triton X-100. The attached cells were then scraped down in the lysis buffer and collected into a separate 1.5 ml Eppendorf tube. The insoluble pellets from the conditioned culture media were combined with the lysed cells in the lysis buffer. The cell lysates were incubated for 90 minutes at 4^º^C and then centrifuged at 15,000 rpm for 15 minutes to remove cell debris.

### SDS–PAGE and western blotting

Protein concentrations of cell lysates were determined by bicinchoninic acid microprotein assays (Pierce Inc., Rockford, IL, USA) against albumin standards. Equal protein samples (20 μg) were prepared for SDS–PAGE in 8x loading buffer containing 0.25 M Tris (pH 6.8), two mM DTT, 8% SDS, and 0.02% bromophenol blue. Each sample was subjected to SDS–PAGE electrophoresis on a 4–20% precast-gels (Bio-Rad) and then transferred onto PVDF membranes. The membranes were blocked in 5% milk for 1h and then incubated with primary antibodies (1/1000) overnight. The secondary antibodies, anti-rabbit or anti-mouse IgG conjugated phosphatase, were then added for 1h at room temperature afterward. The blots were then washed with TBST, and immunoreactive bands were visualized by developing with biotin, avidin-conjugated alkaline phosphatase, Nitro blue tetrazolium, and 5-Bromo-4-chloro- 3-indolyl phosphate (BCIT/NBT) developer (KPL, Gaithersburg, MD, USA). A 250 kDa to 14 kDa rainbow molecular weight marker (RPN800E, GE Healthcare, Bio-Sciences, Pittsburgh, PA, USA) was loaded in the first well of the electrophoretic gel to estimate the molecular weight of each band. Quantitative evaluation of protein levels was performed via computer-assisted densitometric scanning (NIH ImageJ, version 1.6 software).

### Statistical analysis

All experiments were done independently on different days at least three times. N2a cells were seeded at least three separate times, and each experiment was treated independently with the chemicals accordingly. As for the primary culture, three separate batches of n = 3 (representing three biological replicates) were obtained from the company on different days. All biological replicates treatments were done at different times and days. Data are plotted as mean ± SEM. Statistical analysis was performed with one-way ANOVA Tukey’s Test. For multiple comparisons, one-way ANOVA followed by Bonferroni’s post-hoc test was performed. *p<0.05, **p<0.01, ***p<0.001, **** p<0.0001, ns: non-significant. GraphPad Prism 8.0 (GraphPad, La Jolla, CA).

## Results

**Fig 1:**
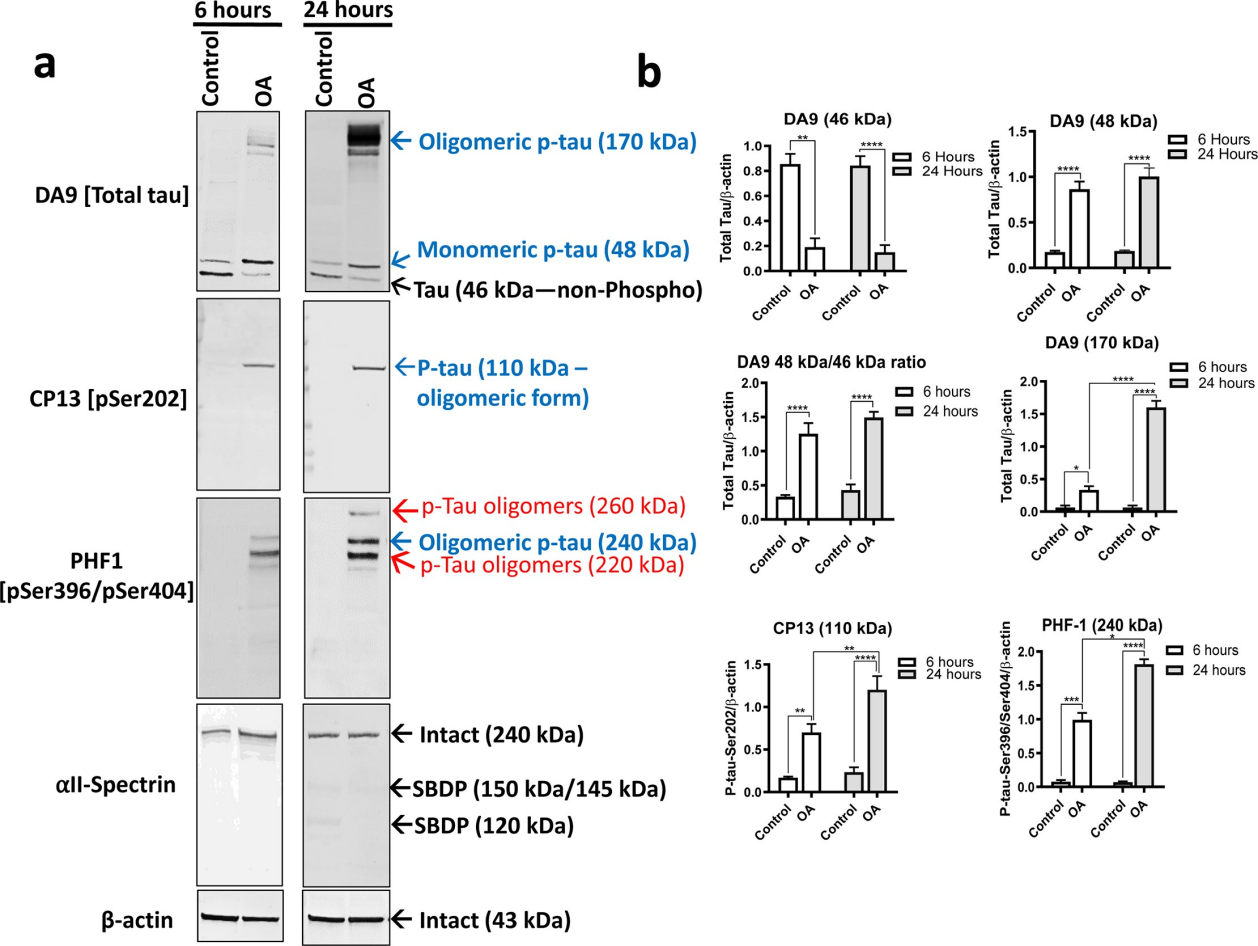
OA induced tau hyperphosphorylation and oligomerization at different time points in mouse neuroblastoma N2a cells. (a). Immunoblots of N2a cells extracted protein (20 μg) using total and phospho-tau antibodies: DA9 (a.a. 102–140), CP13 (pSer202), and PHF-1 (pSer396/pSer404). ⍺II-Spectrin antibody was used to assess neuronal apoptotic pathway activation through monitoring intact spectrin (240 kDa), SBDP150/145 (calpain activation), and SBDP120 (caspase-3 activation). Different tau species are pointed with colored arrows. Blue arrows present monomeric p-tau (48 kDa), and oligomeric p-tau (110 kDa, 170 kDa, and 240 kDa). Red arrows on PHF-1 points on two minor bands of oligomeric p-tau (220 kDa and 260 kDa). Black arrows show non-phospho tau band (46 kDa). SNJ-1945 (abbreviated as S; a calpain inhibitor, 100 μM) and Z-DCB (abbreviated as Z; a caspase-3 inhibitor, 60 μM) were added for all experimental conditions for 1h before the treatment with OA (100 nM) for 6h or 24h, to prevent apoptosis-mediated proteolysis of tau and ⍺II-Spectrin. A reverse-time course followed OA treatment, and all cells were collected at the same time and conditions. (b). Immunoblots quantification. All data are normalized to β-actin and are expressed as a percentage of control. Data are presented as ± SEM for n = 3. Statistical analysis was performed with one-way ANOVA. For multiple comparisons, one-way ANOVA followed by Bonferroni’s post-hoc test was performed. *p<0.05, **p<0.01, ***p<0.001, ****p<0.0001 and ns: non-significant.

Okadaic acid (OA), a potent PP2A/PP1 inhibitor, induces tau hyperphosphorylation and aggregation [81, 91]. To establish our tauopathy-relevant cell model, mouse neuroblastoma N2a cells were treated with okadaic acid (OA) (100 nM) to induce tau hyperphosphorylation and oligomerization for 6h and 24h (**Fig 1A and 1B**). OA concentration was selected based on previous studies that used similar concentrations optimized on neuronal cell culture [56, 91–93]. Since OA induces apoptosis [94], cell-permeable calpain (SNJ-1945) and caspase-3 (Z-DCB) inhibitors were included in all of our experimental conditions to eliminate modifications resulting from cell metabolism/health [95, 96]. To assess neuronal culture health, the samples were probed with the αII-spectrin antibody. αII-spectrin protein is a key substrate for cysteine proteases associated with necrosis (calpain) and apoptotic (caspase-3) cell death [97]. Cleavage of αII-spectrin by calpain shows major spectrin breakdown products of molecular weight 150 kDa (SBDP150), and 145 kDa (SBDP145), while caspase-3 activation produces a major cleavage product of 120 kDa (SBDP120) detectable by western blotting [97, 98].

Control samples probed with αII-spectrin showed a high molecular weight 240 kDa band (intact αII-spectrin), while SBDPs were absent, suggesting a healthy metabolism and neuronal culture **(Fig 1A)**. Western blots were analyzed with total tau monoclonal antibody DA9 (a.a. 102–140), and monoclonal phospho-tau (p-tau) antibodies, including CP13 (pSer202), and PHF-1 (pSer396/pSer404) (**Table 1**). The β-actin antibody was used to evaluate the evenness of loading the protein extracts. Untreated control showed that the total tau antibody DA9 detected tau protein bands at 46 kDa and 48 kDa (**Fig 1A**). The intensity of the band at 46 kDa was higher compared to the band at 48 kDa in control samples (**Fig 1A**).

Treatment with OA (100 nM) for 6h and 24h showed a dramatic decrease in levels of the 46 kDa and increased levels of 48 kDa with DA9 antibody. The 46 kDa and 48 kDa bands may be different tau isoforms, as reported in previous studies [99]. These bands might also include phosphorylated (p-tau) and non-phosphorylated tau (tau) along with the different tau isoforms. In our study, since OA caused a shift in these bands, the 46 kDa band was assigned as tau and the 48 kDa band as p-tau. Treatment with OA showed high molecular weight (HMW) band clusters at 170 kDa, probed with DA9 antibody (a.a 102–145) for 6h (p<0.05) and 24h (p<0.0005). These (HMW) bands may represent the formation of tau oligomers as they were not observed in control cells and only with OA-treated cells. Because the tau phosphorylation and the formation of HMW bands were observed relatively at higher levels in OA treatment for 24h compared to the 6h, the 24h treatment was selected as our tauopathy model (**Fig 1A and 1B**).

Probing with CP13 (pSer202) antibody did not show any detectable bands of tau protein in control samples **(Fig 1A)**. However, with OA treatment, CP13 showed a high molecular weight (HMW) band formed at 110 kDa (x2 size of monomeric tau) with 6h and 24h (**Fig 1A and 1B**). Probing with the PHF-1 antibody (pSer396/pSer404) did not show any tau band with control samples (**Fig 1A and 1B**). Treatment with OA for 6h and 24h showed HMW cluster of bands at 220 kDa, 240 kDa, and 260 kDa with PHF-1 (**Fig 1A and 1B**). Notably, the 260 kDa band (red arrow) (**Fig 1A**) was only detectable with OA treatment for 24h (PHF-1). Low molecular weight monomeric tau (LMW-MT) bands were not detected with either CP13 or PHF-1. It should be noted that the DA9 antibody recognizes total tau epitopes from aa. 102–140. Thus, to identify the 48 kDa tau species detected with DA9, the phospho-tau antibody needs to recognize the same epitope. It possible that LMW-MT might be phosphorylated at sites other than Ser202/Ser396/Ser404, and oligomerized into the different HMW tau species detected at 110 kDa, 170 kDa, 220 kDa, 240 kDa, and 260 kDa. Using RZ3(pThr231) and AT270(pThr181), LMW-MT at 48 kDa, and 55 kDa were detected with OA treatment, respectively **(S1 Fig, left panel, OA lane)**. Collectively, these data strongly suggest that OA treatment caused protein phosphatase inhibition inducing the formation of LMW and HMW tau bands, immunoreactive at pSer202 (CP13, 110 kDa), pSer396/pSer404 (PHF-1, 220/240/260 kDa), RZ3 (pThr231, 48 kDa) and AT270 (pThr181, 55 kDa).

### Screening of tau kinase inhibitors on OA-induced tau hyperphosphorylation and oligomerization in N2a neuronal culture

**Fig 2:**
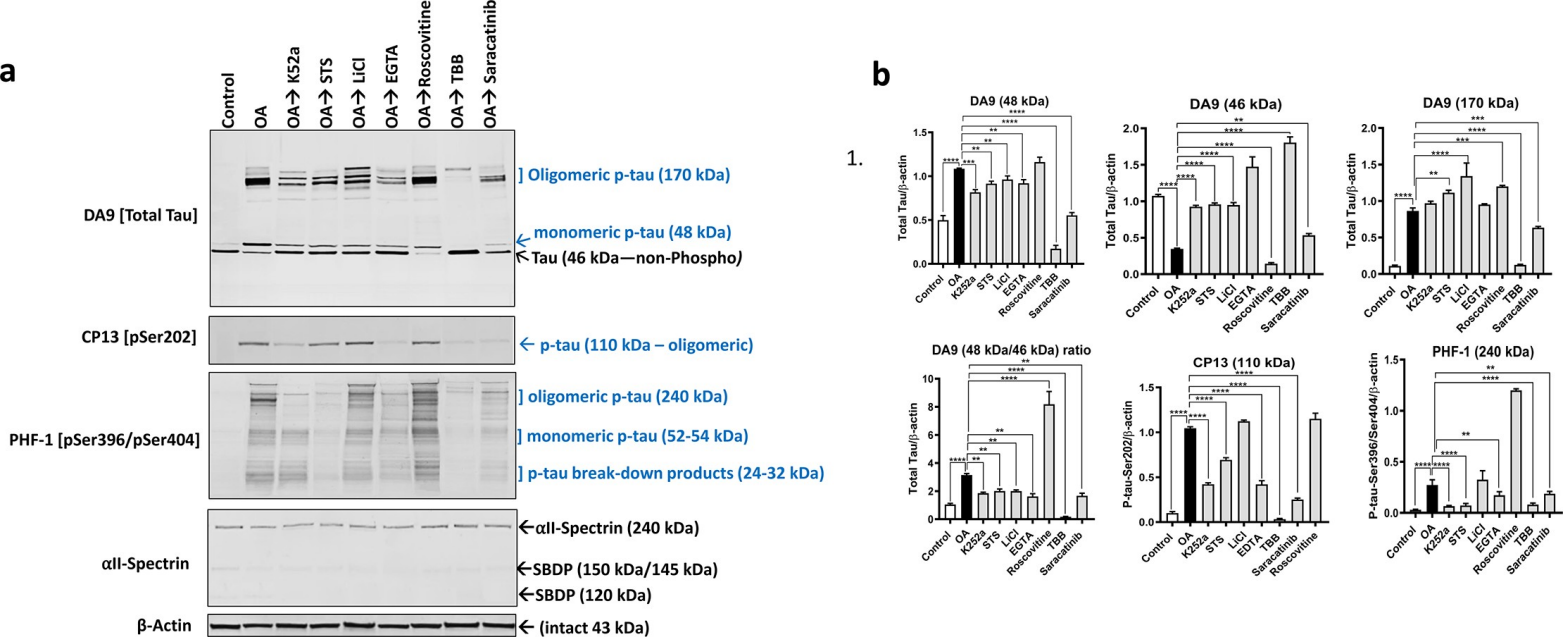
Screening of protein kinase inhibitors on OA-induced Tau hyperphosphorylation and oligomerization in N2a cells. (a). Immunoblots of N2a cells extracted protein (20 μg) using phospho-tau antibodies (CP13, PHF-1), total tau (DA9), and αII-Spectrin. αII-Spectrin was probed to assess neuronal cell injury monitored with SBDP145/150 and SBDP120. Kinase inhibitors effect on OA-induced tau bands (100 nM) was monitored by evaluating the levels of monomeric (48 kDa) and oligomeric p-tau immunoreactivity (110 kDa, 170 kDa, and 240 kDa; blue arrows), total tau, and non-phospho tau (46 kDa; black arrows). Phosphorylated tau break-down products are shown with PHF-1 immunoblot. For all experimental conditions, S (a calpain inhibitor) and Z (a caspase-3 inhibitor) were added for 1h to before the addition of OA for 24h followed by 6h incubation with the kinase inhibitors. The concentrations used for each protein kinase inhibitor are mentioned in materials and methods, cell treatment section. β-actin was probed as a loading control. All experimental conditions were collected and analyzed at the same time. (b). Immunoblots quantification. All data are normalized to β-actin and are expressed as a percentage of control. Data are presented as ± SEM for n = 3. Statistical analysis was performed with one-way ANOVA. For multiple comparisons, one-way ANOVA followed by Bonferroni’s post-hoc test was performed. *p<0.05, **p<0.01, ***p<0.001, ****p<0.0001 and ns: non-significant.

To screen for protein kinase inhibitors as drug candidates for inhibition of OA-induced hyperphosphorylation and oligomerization, mouse neuroblastoma N2a cells were pretreated with OA for 24h followed by treatment with protein kinase inhibitors for 6h. The positive control included only OA treated cells for 24h. Protein kinase inhibitors used included: LiCl (10 mM), AR-A014418 (AR) (60 μM), A-1070722 (A107)(60 μM), K252a (10 μM), STS (0.5 μM) 4,5,6,7-tetrabromobenzotriazole (TBB) (60 μM), Roscovitine (60 μM), Saracatinib (100 μM), cyclosporine A (CsA) (60 μM), and EGTA (5 mM) (**Table 1; Fig 2A and 2B).** All conditions were pretreated with SNJ-1945 (calpain inhibitor, abbreviated as S; 60 μM) and Z-DCB (caspase inhibitor, abbreviated as Z; 100 μM) to minimize apoptotic pathway activation (calpain and caspase-mediated proteolysis) [95, 100] (**Table 1**).

### Casein kinase II (CKII) inhibitor: 4,5,6,7-tetrabromobenzotriazole (TBB)

**Fig 3:**
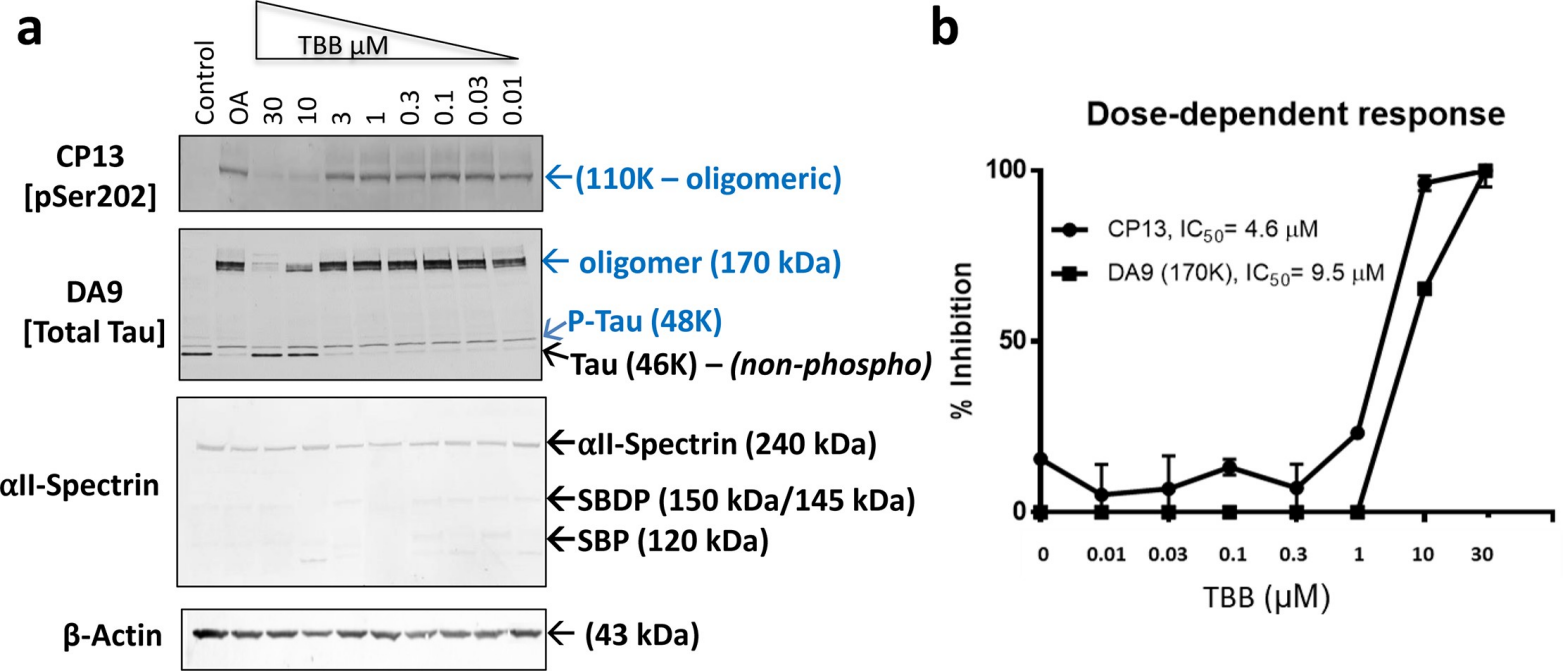
Dose-response of TBB on OA-induced tau hyperphosphorylation and oligomerization in N2a cells. N2a cells were pre-treated with OA for 24h followed by treatment with different concentrations of TBB for 6h, as indicated in the Figure. (a) Immunoblots of cell extracted proteins using phospho-tau antibodies, including CP13 (pSer202), and total tau DA9 (a.a. 102–140). Blue arrows represent monomeric and oligomeric p-tau (48 kDa, 110 kDa, and 170 kDa). αII-Spectrin antibody used to monitor SBDPs with the increasing concentrations of TBB. The β-actin antibody was used as a loading control. All conditions included SNJ-1945 (calpain inhibitor) and Z-DCB (caspase inhibitor). (b) TBB dose-response treatment line chart. TBB concentration (in micromolar) is shown on the X-axis, and the inhibition percentage is presented on the Y-axis. The control sample values were designated as the standard response. The X-axis concentration values are logarithm-transformed to fit a straight line. The half-maximal inhibitory concentration (IC_50_) was used to measure the effectiveness of TBB in inhibiting OA-induced tau hyperphosphorylation and oligomerization. GraphPad Prism was used to calculate the IC_50_ (for DA9 and CP13 antibodies) and are presented on the figure. The statistical analysis was performed with one-way ANOVA, followed by Bonferroni's post-hoc test. *p<0.05, **p<0.01, ***p<0.001. Data are presented as ± SEM for n = 3.

Since aberrant CKII has been reported in AD [101], TBB, a cell-permeable CKII inhibitor, was selected for the study. Total tau DA9 showed that TBB abolished the 48 kDa band (p-tau) and the HMW 170 kDa band (tau oligomers), and significantly increased (p<0.0001) levels of the 46 kDa (non-phospho tau) by 85%, compared to OA treatment alone (**Fig 2A and 2B, Table 3**). CP13 (pSer202) antibody showed that TBB eliminated the OA-induced 110 kDa band (oligomeric p-tau). Similarly, the PHF-1 antibody (pSer396/pSer404) showed that TBB fully inhibited the formation of 240 kDa (HMW bands-oligomeric tau) (**Fig 2A and 2B, Table 3**). As a selective casein kinase II (CKII) inhibitor, TBB showed robustness in inhibiting both OA-induced tau hyperphosphorylation and oligomerization. Thus, the aim was to evaluate the TBB dose-response effect on N2a neuronal culture. Thus, N2a cells were treated with OA for 24h followed by treatment with various concentrations of TBB (10 nM, 30 nM, 100 nM, 300 nM, 1 μM, 3 μM, 10 μM, and 30 μM) for 6h (**Fig 3A and 3B**). The result shows that treatment with ten micromolars of TBB resulted in a 50% reduction of the 110 kDa (oligomeric p-tau form; CP13), 48 kDa, and 170 kDa bands (monomeric and oligomeric p-tau, DA9) (**Fig 3A and 3B**). Increasing the concentration of TBB up to 30 μM caused a 90% reduction of 48 kDa (monomeric p-tau, DA9), 170 kDa (oligomeric p-tau, DA9) and 110 kDa (oligomeric tau form, CP13) (**Fig 3A and 3B**). As for assessing neuronal culture integrity, the intact αII-spectrin band was detected at 240 kDa, and no SBDP150/145 or SBDP120 was observed with the TBB treated conditions suggesting a healthy culture.

**Table 3:** Composite effects of kinase inhibitors on OA-induced tau hyperphosphorylation in N2a cells.

Inhibitor	Inhibition %		
	CP13 (110 kDa)	PHF-1 (240 kDa)	DA9 (170 kDa)
K252a	62	70	40
STS	32	82	-10
LiCl	-14	-9	-12
EGTA	90	85	55
CsA	98	90	**100**
Roscovitine	-11	-53	-22
**TBB**	**100**	**100**	**100**
Saracatinib	70.5	46	45
A107	23	0	13
AR	0	0	36

A negative sign correspond to an adverse effect. Bold corresponds to maximal inhibition at the tested epitope.

#### Calcineurin inhibitor: Cyclosporin A (CsA)

Cyclosporin A (CsA) has been reported to inhibit the calcineurin phosphatase activity (PP3) and CaMKII by blocking the Ca++ mitochondrial permeability [102] (Table 1). Thus, CsA was selected in this study as a calcium-dependent kinase inhibitor to assess its effect on OA-induced tau hyperphosphorylation and oligomerization. Notably, CsA abolished the 48 kDa and 170 kDa monomeric and oligomeric p-tau of DA9, respectively (**S1 Fig**). Moreover, CsA reduced the p-tau band at 110 kDa of CP13 (oligomeric p-tau band; p<0.0001), and 240 kDa of PHF-1 (oligomeric p-tau band) (**S1 Fig, Table 3**).

RZ3 antibody showed a complete reduction of the 48 kDa band (monomeric p-tau) when cells were treated with CsA (**S1 Fig**). As for the AT270 antibody, OA-treated samples showed a band detected at 55 kDa (monomeric p-tau), which was abolished when neurons were treated with CsA. The 48 kDa and 55 kDa OA-induced bands detected by RZ3 and AT270, respectively, indicate that tau can have various levels of phosphorylation, depending upon the epitope tested. β-actin protein levels remained even in all experimental conditions. Blotting with the αII -spectrin antibody did not show any significant changes of the 240 kDa band (intact form). Moreover, SBDP150/145 or SBDP120 immunoreactive bands were not detected in all of the treated samples, indicative of a healthy metabolism.

#### Calcium chelator: EGTA

Another calcium-dependent kinase inhibitor, EGTA, was used as a calcium-chelating agent. EGTA has a lower binding affinity for Mg^++^ relative to EDTA, making it more selective for Ca^++^ ions [103]. Total tau DA9 showed that EGTA treatment (with calpain and caspase inhibitors; S+Z) caused a 25% reduction of the 48 kDa band (p-tau), an 85% increase of the 46 kDa band (non-phospho-tau) and 55% reduction of the 170 kDa band (oligomeric p-tau form), compared to OA treatment alone (**Fig 2A and 2B and Table 3**). EGTA caused a 90% reduction of the 110 kDa band (oligomeric tau; CP13) and an 85% reduction of 240 kDa (oligomeric tau; PHF-1) (**Fig 2A and 2B and Table 3**). As for apoptotic pathway activation, αII-spectrin antibody did not show any effect on the 240 kDa band (intact form).

#### Glycogen synthase kinase-3 (GSK-3) inhibitors: LiCl, A-1070722, and AR-1014418

The effects of small molecule GSK-3 inhibitors, LiCl, A-1070722 (abbreviated as A-107), and AR-1014418 (abbreviated as AR), were tested on OA-induced tau hyperphosphorylation and oligomerization. LiCl showed an opposite effect in N2a cell treatment by increasing levels of 110 kDa band (oligomeric form, CP13; -14%) and levels of 240 kDa band (oligomeric p-tau, PHF-1; -9%) (**Fig 2A and 2B and Table 3**). As for total tau DA9 antibody, LiCl also showed an opposite effect by increasing the 48/46 kDa (p-tau/non-phospho-tau) band ratio by 20%, and the 170 kDa oligomeric tau band by 12%.

Treatment with AR did not show a statistically significant effect on the 240 kDa band (oligomeric p-tau; PHF-1) or 110 kDa band (oligomeric p-tau form; CP13) (**S2 Fig, Table 3**). Probing with total tau DA9 showed that AR treatment caused an adverse effect by increasing the 48 kDa/46 kDa ratio (monomeric tau form; -50%) and reducing the 170 kDa oligomeric form band by 36% (**S2 Fig, Table 3**). Another potent GSK-3 inhibitor, A-107 (Ki = 0.6 nM for GSKα and GSK-3β) [69], was selected for the study. OA followed by A-107 treatment showed a 23% reduction of 110 kDa oligomeric p-tau band (CP13), and non-significant, but a partial reduction of the 240 kDa oligomeric p-tau form (PHF-1) compared to OA treatment alone (**S2 Fig, Table 3**). Probing with total tau DA9 showed with A-107 treatment, a 13% reduction of the 170 kDa band (DA9), and did not show a statistically significant effect on the 48 kDa band (**S2 Fig, Table 3**). As for caspase-3, calpain, and cell injury activation, αII-spectrin did not show SBDP 150/145 or SBDP120 post-treatment, indicative of a healthy neuronal culture.

#### Src/Fyn kinase inhibitor: Saracatinib

In the current study, Saracatinib was selected to investigate the role of the Fyn kinase function on the tauopathy-relevant cell-based model [24]. Probing with DA9 (a.a. 102–140) antibody, Saracatinib resulted in a 40% reduction in the 48 kDa (monomeric p-tau), a 20% increase in the 46 kDa (non-phospho tau) bands and 45% reduction of the 170 kDa band (oligomeric p-tau form) (**Fig 2A and 2B, Table 3**). Saracatinib treatment reduced (p<0.0001) the 110 kDa oligomeric p-tau band of CP13 (75%; CP13) and produced a 46% reduction in immunoreactivity of the 240 kDa (oligomeric p-tau form band of PHF-1) (**Fig 2A and 2B, Table 3**). As for assessing cell integrity, intact spectrin (240 kDa) levels remained constant, and SBDP150/145 and SBDP120 levels were not significantly altered.

#### Pan kinase inhibitor: K252a and STS

K252a is a non-selective cell-permeable protein kinase inhibitor, inhibiting protein kinase C (PKC; IC_50_ = 32.9 nM), Ca^2+^/calmodulin-stimulated phosphodiesterases (IC_50_ = 1.3–2.9 μM), serine/threonine protein kinases (IC_50_ = 10–20 nM), myosin light-chain kinase (MLCK; K_i_ = 20 nM), receptor tyrosine kinases, and inhibiting the carcinogenic properties of MET oncogene [104, 105]. K252a is an analog of staurosporine (STS) and has a broad spectrum of protein kinases inhibition, neuroprotection properties, and improvement in psoriasis in vivo (Table 1) [72]. In this study, K252a and STS treatments similarly showed a 30% increase in the 46 kDa (monomeric non-phosphorylated tau) and a 35% decrease at 48 kDa (monomeric p-tau) compared to OA, with DA9 antibody (**Fig 2A and 2B, and Table 3**). K252a treatment caused a 40% reduction of 170 kDa (DA9; oligomeric p-tau) compared to OA treatment alone (**Fig 2A and 2B, and Table 3**). For p-tau detection, probing with the CP13 antibody showed a 60% and a 32% reduction in the 110 kDa (oligomeric p-tau form) with K252a and STS treatment correspondingly. PHF-1 showed a 70% and 80% reduction in levels of 240 kDa (oligomeric form) with K252a and STS treatment, respectively (**Fig 2A and 2B, Table 3**). αII-spectrin immunoreactive bands (intact-240 kDa, SBDP150/145, and SBDP120) did not show a statistically significant difference compared to control values.

#### CDK5 inhibitor: Roscovitine

Roscovitine is a cyclin-dependent kinase 5 (CDK5) inhibitor that acts through direct competition at the ATP-binding site [75]. Roscovitine showed an adverse effect of increasing levels of oligomeric tau detected at 170 kDa (-51%; DA9), 110 kDa (-11%; CP13), and 240 kDa (-53%; PHF-1) compared to OA treatment alone (**Fig 2A and 2B, Table 3**). Roscovitine showed partial, but a statistically non-significant decrease in the 48 kDa (monomeric p-tau) band of DA9, compared to OA treatment alone. The αII-spectrin antibody did not show a statistically significant difference of intact form (240 kDa), SBDP150/145, and SBDP120 compared to control values.

### Baseline and OA-induced tau hyperphosphorylation and oligomerization: Effects of various kinase inhibitors treatments in rat primary cerebrocortical neuronal (CTX) culture

To further expand our experimental paradigm in a culture-based model suitable for drug candidate screening, the effectiveness of the protein kinase inhibitors was investigated on differentiated rat primary cerebrocortical neuronal (CTX) cultures. Therefore, CTX cells were pretreated with or without OA 24h (100 nM) (**Table 1**) followed by treatment with protein kinase inhibitors for 6h. Calpain and caspase-3 inhibitors, SNJ-1945 and Z-DCB, respectively, were added to all experimental conditions to prevent cell death-mediated proteolysis of tau as a potential confound. culture health.

**Fig 4:**
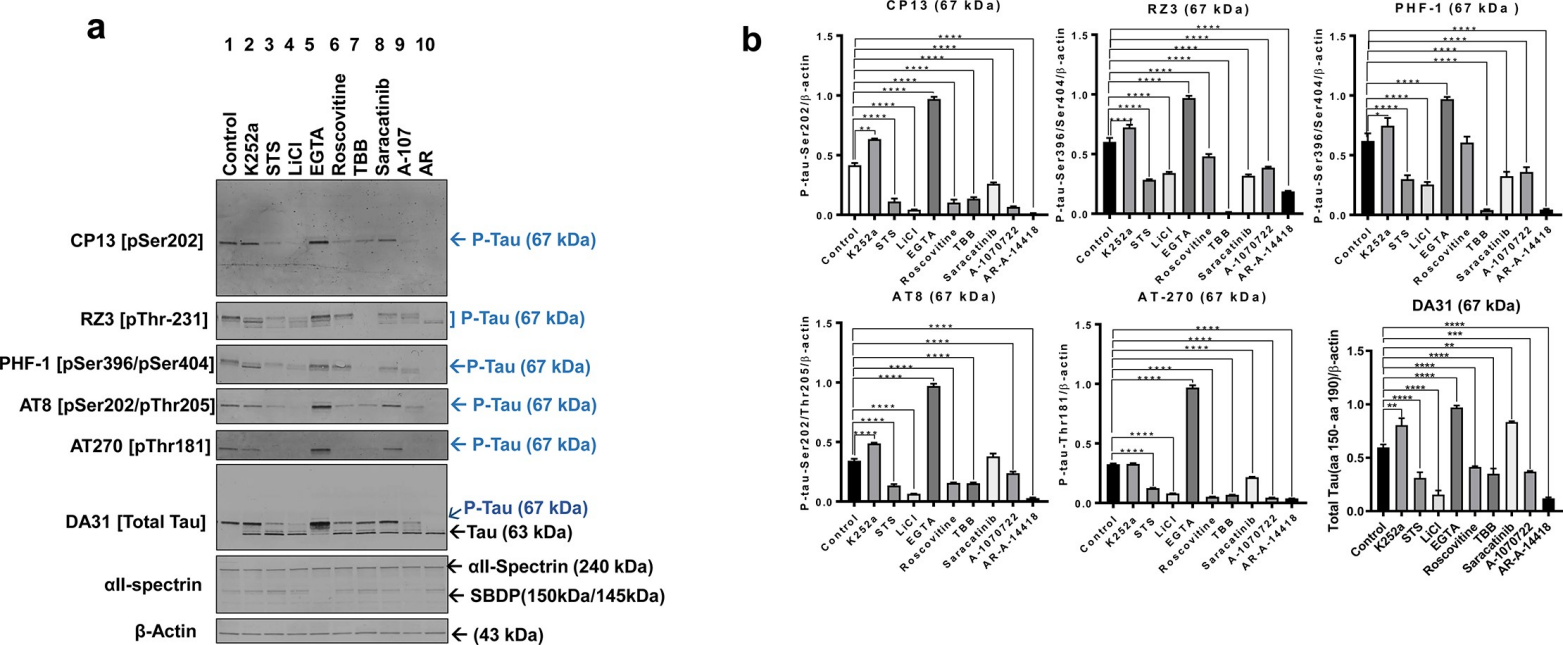
Screening of protein kinase inhibitors on physiologically phosphorylated tau in rat primary cerebrocortical neuronal culture. Rat primary cerebrocortical neuronal differentiated cultures (CTX) at 15 DIVs, were treated various protein kinases inhibitors, including K252a (30 μM), STS (20 μM), LiCl (10 μM), EGTA (5 mM), Roscovitine (60 μM), Saracatinib (100 μM), TBB (30 μM) and A-107 (20 μM), AR (60 μM) for 6h. Calpain and caspase inhibitors (S+Z) were added to all experimental conditions for 1h before the protein kinase inhibitor treatments. Cell lysates were analyzed on western blots using twenty micrograms of protein. (a) Immunoblots of cell lysates analyzed for phosphorylated tau at the epitopes CP13 (pSer202), PHF-1 (pSer396/404), AT8 (pSer202/pThr205), RZ3 (pThr231), and AT270 (pThr181). Total tau was probed with DA31 (a.a. 150–190) antibody. DA31 blot showed two distinctive tau bands (63 kDa, non-phospho tau, and 67 kDa, p-tau) following kinase inhibitors treatment. SBDP145/150 and SBDP120 were analyzed with the αII-spectrin antibody. Different lanes are numbered at the top of each label in the figure. (b) Immunoblot quantification of basal tau phosphorylation. Ratios of phospho-epitope levels over β-actin ± SD are represented as a percentage. Statistical analysis was performed with one-way ANOVA. For multiple comparisons, one-way ANOVA followed by Bonferroni’s post-hoc test was performed. *p<0.05, **p<0.01, ***p<0.001 and ****p<0.0001. n = 3 per condition.

**Fig 5:**
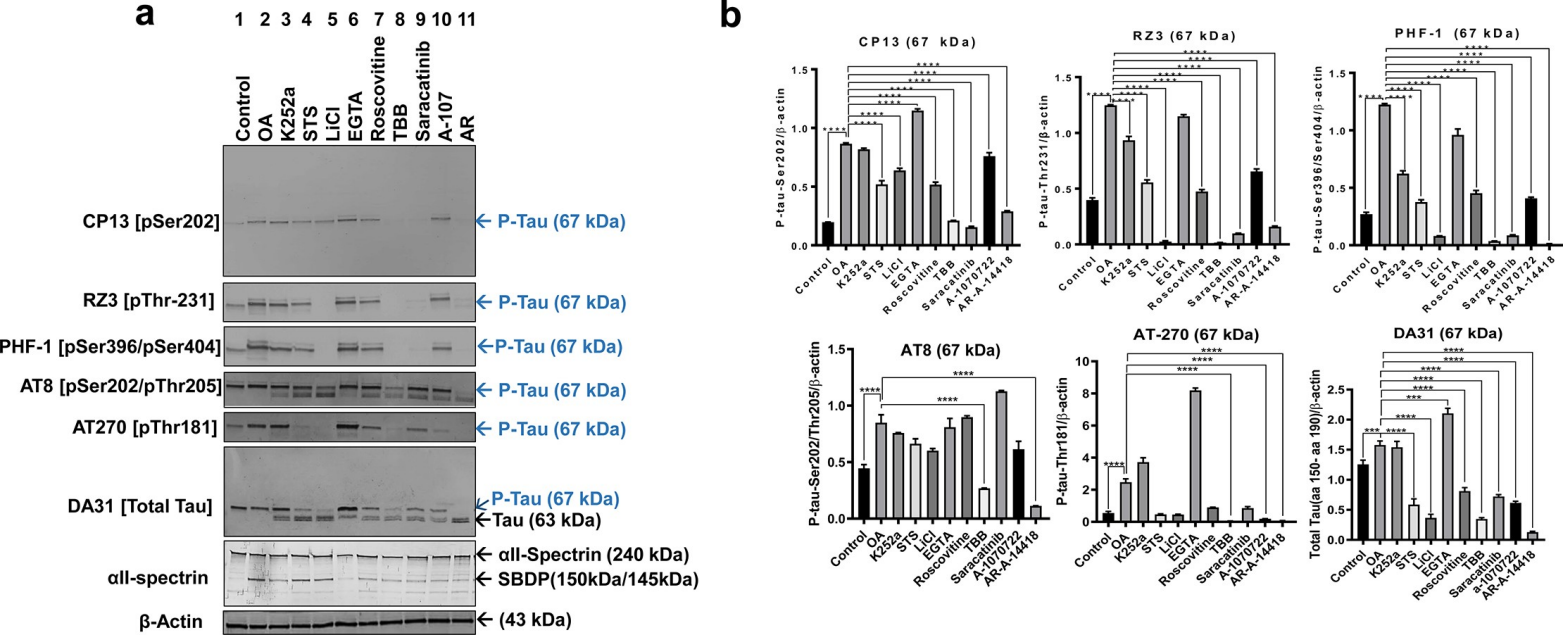
Effect of protein kinase inhibitors on OA-induced tau hyperphosphorylation in rat primary cerebrocortical neuronal culture. Rat primary cerebrocortical neuronal differentiated cultures (CTX) at 15 DIV were treated with OA (100 nM) for 24h followed by protein kinases inhibitors for 6h. The concentrations of kinase inhibitors are the same as the ones mentioned in **Fig 4**. CTX cultures were treated with S and Z for 1h before any treatment to prevent apoptotic pathway-mediated tau proteolysis. (a). Immunoblots of cell lysates analyzed for phosphorylated tau at the epitopes CP13, PHF-1, AT8, RZ3, AT270. Total tau was probed with the DA31 antibody. With DA31 blot, the 63 kDa band is referred to as monomeric non-phospho tau and the 67 kDa as monomeric p-tau species. Spectrin Break down products (SBDPs) were monitored with the αII-spectrin antibody. (b) Immunoblot quantification of OA-induced tau phosphorylation. Ratios of phospho-epitope levels over β-actin ± SD are represented as a percentage. Statistical analysis was performed with one-way ANOVA. For multiple comparisons, one-way ANOVA followed by Bonferroni’s post hoc test was performed. *p<0.05, **p<0.01, ***p<0.001 and ****p<0.0001. n = 3 per condition.

CTX control cultures showed normal cell bodies and healthy neurites, including axons and dendrites. Untreated control samples showed basal levels of phosphorylated tau (67 kDa) detected by total and p-tau antibodies, including: DA31 (a.a.150-190), CP13 (pSer202), RZ3 (pThr231), PHF-1 (pSer396/pSer404), AT8 (pSer202/pThr205), and AT270 (pThr205) (**Fig 4A and 4B, lane 1**). Treatment with OA for 24h caused a dramatic increase of 67 kDa band (monomeric p-tau) at multiple phospho-tau epitopes (CP13: 9x, RZ3: 9.8x, PHF-1: 13x, AT8: 3x, and AT270: 10x) (**Fig 5A and 5B, lane 2**).

Since the concentration of 30 μM TBB resulted in at least 90% inhibition in N2a cells, the same concentration was used for CTX culture. Treating CTX culture with TBB reduced basal and OA-induced tau phosphorylation (67 kDa) at CP13 (-OA: 91%, +OA: 98%), RZ3 (-OA: 100%, +OA: 100%), PHF-1 (-OA: 100%, +OA: 100%), AT8 (-OA: 91%, +OA: 100%), and AT270 (-OA: 100%, +OA: 100%) compared to OA treatment alone (**Fig 4A and 4B, lane 7 and Fig 5A and 5B, lane 8, Table 4**). Total tau DA31 (a.a. 150–190) antibody detected immunoreactive bands at 63 kDa and 67 kDa with the different kinase inhibitor treatments. The decreased electrophoretic mobility of the 63 kDa might correspond to the lower levels of p-tau protein induced by the protein kinase inhibitors; thus, this band was assigned as non-phospho-tau. TBB treatment reduced the phospho-tau band at 67 kDa (-OA: 41%, +OA: 91%), and increased non-phospho tau band at 63 kDa (-OA: +53%, +OA: +81%) (**Fig 4A and 4B, lane 7 and Fig 5A and 5B, lane 8, Table 4**).

**Table 4:** Composite effects of kinase inhibitors on basal and OA-induced tau hyperphosphorylation in rat primary cerebrocortical neuronal cells.

Inhibitor		Inhibition %					
		CP13	RZ3	PHF-1	AT8	AT270	DA31

K252a	- OA	35	45	61	10	17	41
	+ OA	41	37	63	0	0	45
STS	- OA	86	83	55	88	**100**	79
	+ OA	63	81	89	12	**100**	77
**LiCl**	- OA	94	89	98	**100**	**100**	93
	+ OA	50	100	100	81	**100**	96
EGTA	- OA	-51	-63	-22	-64	-68	-73
	+ OA	-12	-22	-13	-5	-63	-69
Roscovitine	- OA	85	42	63	91	**100**	32
	+ OA	63	91	81	0	18	29
**TBB**	- OA	91	**100**	**100**	91	**100**	41
	+ OA	98	**100**	**100**	**100**	**100**	91
**Saracatinib**	- OA	41	81	52	0	0	5
	+ OA	**100**	**100**	**100**	0	84	20
A-107	- OA	92	79	65	82	**100**	80
	+ OA	36	70	85	21	**100**	55
**AR**	- OA	**100**	89	**100**	**100**	**100**	**100**
	+ OA	89	**100**	**100**	**100**	**100**	**100**
CsA	- OA	90	91	89	95	92	95
	+ OA	33	26	0	86	11	28

A negative sign correspond to an adverse effect. Bold corresponds to maximal inhibition at the tested epitope. The -/+ OA indicates either the presence or absence of okadaic acid compound. The densitometric intensity of 67 kDa band from **Fig 4** and **Fig 5** was used for calculating the percentage of inhibition.

In contrast to N2a cells, LiCl reduced basal and OA-induced tau phosphorylation (monomeric p-tau, 67 kDa) in CTX culture at CP13 (-OA: 94%, +OA: 50%), RZ3 (-OA: 89%, +OA: 100%), PHF-1 (-OA: 98%, +OA: 100%), AT8 (-OA: 100%, +OA: 81%), AT270 (-OA: 100%, +OA: 100%) and total tau DA31 (-OA:93%, +OA: 96%) (**Fig 4A and 4B, lane 4 and Fig 5A and 5B, lane 5, Table 4).** AR also abolished the 67 kDa band with basal and OA-induced tau hyperphosphorylation (**Fig 4A and 4B, lane 10 and Fig 5A and 5B, lane 11, Table 4**). With total tau DA31 (a.a. 150–190), LiCl and AR eliminated the 67 kDa (monomeric p-tau) and substantially increased the 63 kDa band (non-phospho tau). Treating CTX neuronal culture with A107 also showed a substantial inhibition of 67 kDa (-OA and +OA) band with CP13 (-OA: 92%, +OA: 36%), RZ3 (-OA: 79%, +OA: 70%), PHF-1 (-OA: 65%, +OA: 85%), AT8 (-OA: 82%, +OA: 21%), AT270 (-OA: 100%, +OA: 100%), and total tau DA31 (-OA: 80%, +OA: 55%), compared to OA treatment alone (**Fig 4A and 4B, lane 9 and Fig 5A and 5B, lane 10, Table 4**). As for Roscovitine treatment, in contrast to N2a neuronal treatment, the 67 kDa band was reduced considerably at CP13 (-OA: 85%, +OA: 63%), RZ3 (-OA: 42%, +OA: 91%), PHF-1 (-OA: 63%, +OA: 81%), and total tau DA31 (-OA, +OA: ~30%). However, Roscovitine did not show a statistically significant effect on OA-induced tau phosphorylation at AT8 and AT270 (**Fig 4A and 4B, lane 6 and Fig 5A and 5B, lane 7, Table 4**).

On the other hand, CsA caused a molecular weight shift in the electrophoretic mobility of the 67 kDa to 63 kDa at the sites CP13 (pSer202), RZ3 (pThr231), and DA31 (a.a. 102–145), presumably accounting for the dephosphorylation of tau (**S3 Fig**). Regarding the 67 kDa band, CsA had dramatic inhibition on basal tau phosphorylation at: CP13 (90%), RZ3 (91%), PHF-1 (89%), AT8 (95%), AT270 (92%) and total tau DA31 (67 kDa, 95%) (**S3 Fig, Table 4**). With OA treatment, CsA also showed a considerable immunoreactivity reduction of the 67 kDa band at the epitopes: CP13 (33%), AT8 (86%), and total tau DA31 (28%). CsA had no effect on 67 kDa band at PHF-1, AT270, and RZ3 compared to OA treatment alone (**S3 Fig, Table 4**). Minor oligomeric bands were observed at 240 kDa with PHF-1 antibody in OA treated samples. Based on the αII-spectrin blot, CTX cultures demonstrated intact spectrin (240 kDa) and the absence of any SBDPs, suggesting a healthy metabolism under the experimental conditions. Furthermore, Saracatinib reduced basal and OA-induced tau hyperphosphorylation at: CP13 (-OA: 41%, +OA: 100%), RZ3 (-OA: 81%, +OA: 100%), PHF-1 (-OA: 52%, +OA: 100%), AT270 (-OA: 0%, +OA: 84%) and total tau DA31 (-OA: 5%, +OA: 20%). Saracatinib did not show any significant effect at AT8 phospho-tau epitope (pSer202/pThr205 sites) (**Fig 4A and 4B, lane 8 and Fig 5A and 5B, lane 9; Table 4).**

Treatment with K252a caused substantial inhibition of the 67 kDa band, with basal and OA-induced treatments at CP13 (-OA: 35%, +OA: 41%), RZ3 (-OA: 45%, +OA: 37%), PHF-1 (-OA: 61%, +OA: 63%), and total tau DA31 (-OA: 41%, +OA: 45%) (**Fig 4A and 4B, lane 2, and Fig 5A and 5B, lane 3; Table 4**). K252a did not show any statistically significant inhibition at AT8 and AT270 with both basal and OA-induced tau hyperphosphorylation (**Fig 4A and 4B, lane 2; 5A and 5B, lane 3; Table 4**). Cultures treated with STS showed considerable reduction of basal and OA-induced tau phosphorylation at CP13 (-OA: 86%, +OA: 63%), RZ3 (-OA: 83%, +OA: 81%), PHF-1 (-OA: 55%, +OA: 89%), AT8 (-OA: 88%, +OA: 12%), AT270 (-OA: 100%, +OA: 100%), and total tau DA31 (-OA: 41%, +OA: 45%) (**Fig 4A and 4B, lane 3a and Fig 5A and 5B, lane 4; Table 4**). Unexpectedly, EGTA caused an adverse effect in CTX culture by further enhancing physiological p-tau and OA-induced tau hyperphosphorylation at CP13 (-OA: -51%, +OA: -12%), RZ3 (-OA: -63%, +OA: -22%), PHF-1 (-OA: -22%, +OA: -13%), AT8 (-OA: -64%, +OA: -5%), AT270 (-OA: -68%, +OA: -63%), and total tau DA31 (-OA: -73%, +OA: -69%) (**Fig 4A and 4B, lane 5 and Fig 5A and 5B, lane 6; Table 4**).

Taken all together, treatments with CKII inhibitor TBB, GSK3 inhibitors LiCl and AR, and Src/Fyn Kinase inhibitor Saracatinib showed robust inhibition leading to different reduced basal and OA-induced tau phosphorylation profiles demonstrating the specificity of inhibitors tested in our tauopathy cell-based models. Thus, the kinase inhibitors studied provide targets to reduce or prevent tau hyperphosphorylation and aggregation in tauopathies.

## Discussion

**Fig 6:**
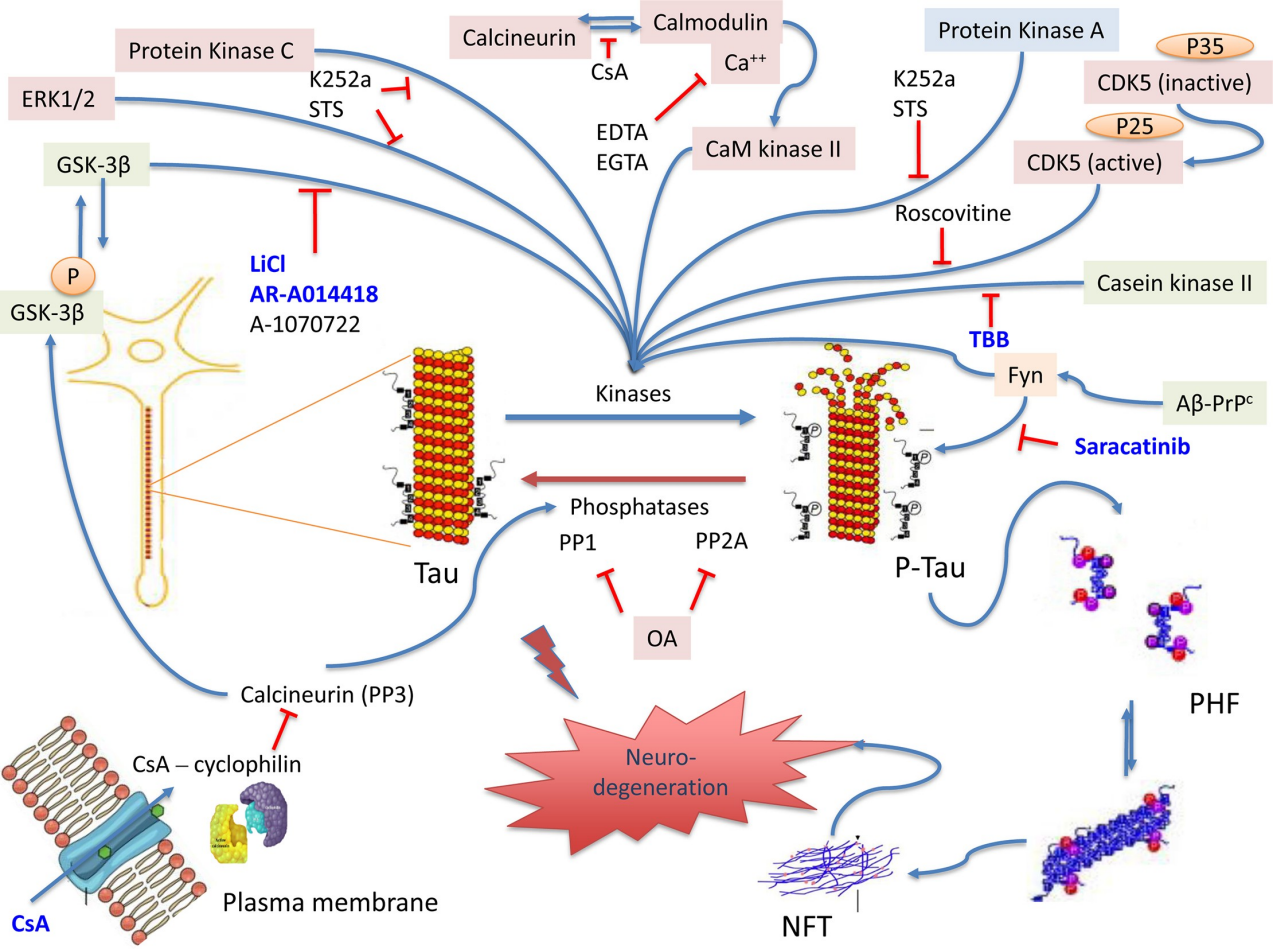
The tauopathy-model and a proposed mechanism for various protein kinase inhibitors intervention. Dephosphorylated tau protein binds the microtubules to maintain it in the polymerized state. Phosphorylation of tau protein by a host of different kinases causes tau to dissociate from the microtubules. Dissociation of tau causes the microtubules to depolymerize. Specific phosphatases dephosphorylate tau allowing the microtubule to re-polymerize again, a physiological process that provides structure and shape to the cytoskeleton of neurons. In tauopathies, imbalances between kinases and phosphatases functions lead to tau hyperphosphorylation at particular pathological sites and a higher tendency to dissociate from the microtubules producing soluble tau aggregates and insoluble paired helical filaments (PHF), that could combine to form neurofibrillary tangles (NFT). NFT is known to be the toxic species in AD and CTE, including other tauopathy diseases, and little is known about their active mechanism of neurodegeneration. OA inhibits the function of crucial tau phosphatases (PP1 and PP2A), leading to activation of tau kinases and tau hyperphosphorylation initiating the pathological processes of tauopathies. One pharmaceutical approach to reverse the mechanism of tauopathies is kinase inhibition. The protein kinase inhibitors selected in this study are indicated in this figure. The inhibitors highlighted in blue are ones that showed a promising effect on our OA-induced cell-based tauopathy model. Microsoft PowerPoint was used to create the artwork.

It has been reported that OA results in robust tau hyperphosphorylation at multiple pathological epitopes in animal and cell culture studies [56, 58, 59, 106–108]. It is widely established that PP2A is the primary enzyme responsible for dephosphorylation of tau protein throughout the brain, controlling all tau phosphorylation sites. PP2A activity is decreased in AD and TBI brains [20, 109]. Therefore, the OA-induced inhibition of PP2A is a highly relevant model to study various tau protein kinase inhibitors as modulators of tau hyperphosphorylation and oligomerization targeting tau pathology **(Fig 6, Table 2)**. Thus, in the present study, OA was used to induce tauopathy hallmarks in mouse neuroblastoma N2a culture and rat primary cerebrocortical neuronal cultures (CTX) as a model to screen for various tau kinase inhibitors as potential drug candidates. The N2a neuronal cultures have been widely used to study mechanisms of neurodegeneration because they are a homogenous culture system convenient to handle and can multiply quickly to produce a tremendous amount of neuron precursor cells [110]. Our CTX culture is fully differentiated neurons, which can provide a model for physiologically relevant cellular events that make neurons uniquely susceptible to disease-associated proteins. The CTX cultures also represent a healthier form of cortical neurons as opposed to cell lines, which are cancerous, in a sense that gene expression in primary cortical culture could represent and mimic the actual *in vivo* expression. Additionally, primary culture has the advantage in portraying the complexity of the central nervous system by better translating into *in vivo* models used for screening pharmaceutical drug candidate compounds [111]. Thus, the use of high-throughput primary culture allowed us to screen multiple drug candidates in a short period, compared to conventional methods, and permit the exposure of novel biological concepts to identify new drug targets for therapeutics.

Western blot analysis showed that OA induced tau hyperphosphorylation and oligomerization at various phospho-tau epitopes in N2a cell culture as a tauopathy model. It is well-known that OA induces apoptosis in human neuroblastoma cells, mouse neuroblastoma, and rat cerebellum neurons [94]. Thus, in our study, the time points were not increased beyond 24h of treatment to avoid tau phosphorylation modifications resulting from proteolysis and neural death. OA caused down-regulation of protein phosphatase and showed the appearance of monomeric (at approximately 46—67kDa) and oligomeric forms of p-tau species (110–240 kDa) immunoreactive to the p-tau-specific antibodies (pSer202, pSer396/404) and total anti-tau (a.a. 102–142). DA9 in the control samples **(Fig 1A)** showed a faint band at 48 kDa and a major band at 46 kDa. Upon the addition of OA to N2a culture, we observed a significant increase in the 48 kDa band and a decrease in the 46 kDa. Both bands are within the range of monomeric tau protein; however, the fact that OA addition caused this shift suggests that these two different bands might not represent only spliced variants of tau protein. It would be expected to observe an increase in both band's phosphorylation levels after OA treatment, even if they were alternatively spliced form of tau.

Moreover, the 63–67 kDa band detected in CTX culture **(Figs 4 and 5)** might be a different tau isoform expressed from the ones in N2a cell culture. Several studies have analyzed the alternatively spliced tau protein using western blotting, showing a distinct profile of molecular weights ranging from 46–67 kDa corresponding to the isoforms 0N3R, 0N4R, 1N3R, 2N3R, 2N4R [112–114]. As described above, any tau post-translational modification, including phosphorylation, glycosylation, deamination, ubiquitination, sumoylation, oxidation, and nitration, can exacerbate tau isoforms [8]. The high-molecular-weight p-tau species (110, 170, 220, 240, and 260 kDa) identified with different antibodies **(Fig 1A)** might be a product of p-tau self-aggregation, including dimers and trimers. It was discovered that any tau isoforms could develop into tau dimers. Two independent tau proteins can associate through cysteine residues via microtubule-binding domain (MBD), creating tau dimers linked covalently by a disulfide bridge, and organized in an anti-parallel direction [115]. There are two distinctive types of dimeric tau: reducible (cysteine-dependent) or non-reducible (cysteine-independent) dimers. Both types were discovered in cell culture and transgenic mice JNPL3 expressing tau’s P301L mutation [116]. The molecular weight for small soluble tau oligomers vary from 120–180 kDa, both for recombinant tau and derived from human tau transgenic mice [116–119]. Tau breakdown products with a mass of dimer and trimer were observed from AD brain synapses [120]. Furthermore, tau oligomers with six to eight tau molecules ranging from 300–500 kDa were identified, potentially produced from soluble dimeric tau [116]. Although the exact mechanism of tauopathy-induced disorders is not yet elucidated, the immunostaining of autopsy brains with anti-p-tau antibodies, including AT8 (pSer202/pThr205), and PHF-1 (pSer396/pSer404) are utilized as a diagnostic method of AD and tauopathy-related diseases [121]. Thus, in our study, the increase in tau phosphorylation detected was identified at these sites as a representation of a tauopathy model. Among all the phospho-tau epitopes studied here, the Thr231 epitope is thought to be associated with the initiation of tau hyperphosphorylation in tauopathies. In contrast, epitopes such as Thr181, Ser202/Thr205, and Ser396/Ser404 are phosphorylated far ahead during the tauopathy process and the progression of the disease [122]. These phosphorylation sites were selected in our study to associate the effectiveness of protein kinase inhibitors with tauopathy-relevant phosphorylation sites.

Previous reports using immunocytochemistry and western blotting showed that in CTX culture, tau is physiologically highly phosphorylated [123]. Our CTX culture showed basal physiological levels of tau phosphorylation in control samples. Multiple tau kinases phosphorylation is generally considered normal. We observed a reduction of p-tau levels with some kinase inhibitors (e.g., STS, LiCl, Roscovitine, A107, and AR) in the absence of OA. There is an equilibrium between tau phosphorylation and dephosphorylation in normal physiological conditions that controls tau’s attachment to microtubules and other biological molecules. Thus, from our data **(Fig 4)**, the reduction of the non-OA induced p-tau by the kinase inhibitors indicate the involvement of the several kinases under physiological conditions.

In contrast to N2a cell culture, only the monomeric form of tau (ranging from 63 kDa– 67 kDa) was observed in CTX culture. One possible reason for such an effect would be that our separation of tau protein by SDS-PAGE was carried out under reducing conditions (Dithiothreitol (DTT) and β-ME) that could minimize tau oligomers to the monomeric form in CTX primary culture. On the other hand, our CTX serum-free neurobasal media contained antioxidants such as glutathione. Thus, the addition of these antioxidants may have blocked the process of tau oligomerization from occurring.

In the N2a and CTX neuronal culture, TBB (CKII inhibitor) provided the most profound reversal of tau phosphorylation and oligomerization at the epitopes pSer202, (CP13), pSer396/pSer404 (PHF-1), pSer202/pThr205 (AT8), pThr181 (AT8), and pThr231 (RZ3). TBB is a selective, cell-permeable, ATP/GTP-competitive inhibitor of casein kinase II (CKII) (IC_50_ = 900 nM for rat liver) [124]. CKII function is aberrant in AD, and its alteration precedes hyperphosphorylated tau accumulation in NFT formation [26]. CKII can phosphorylate tau purified from human brain and neuroblastoma cell line [26, 124–126]. A study has shown that CKII phosphorylates endogenous specific inhibitor (SET), a potent PP2A inhibitor, inducing tau hyperphosphorylation in neurons and animal models, while inhibition of CKII by TBB eliminated this event [127]. Thus, inhibition of CKII by TBB might provide a pharmacological interference for treating tauopathy-related disorders.

Since GSK-3 is a well-known kinase that can phosphorylate tau *in vitro* and *in vivo* and has been proposed as a target for pharmacological intervention [44, 45], three GSK-3 small molecule kinase inhibitors (LiCl, AR, and A-107) were selected to be assessed on OA-induced tauopathy, cell-based model. AR, a thiazole class inhibitor, was shown to decrease insoluble p-tau in the brain stem of transgenic mice overexpressing a mutant human tau protein [43]. In our experiments, AR provided robust suppression of tau hyperphosphorylation in CTX culture at all tau epitopes tested **(Fig 4A and 4B, and Fig 5A and 5B; and Table 4)** and was less effective in N2a cells **(Fig 2A and 2B, and Table 3)**. The effect of AR was more prominent compared to another GSK3 inhibitor, A-107, in CTX primary culture. This effect could be attributed, in part, to the high selectivity and specificity of AR to GSK3β [128] compared to A-107. A-107 display selectivity for both GSK3α and GSK3β (K_i_ = 0.6 nM for both) [129] thereby might dilute the effect of inhibition of GSK3β, which is regarded as the critical kinase in AD [44]. Similarly, a study has shown that hypothermia-induced tau hyperphosphorylation was reduced with AR treatment in human neuroblastoma SH-SY5Y 3R-Tau [108]. In another study, AR protected N2a cell culture against apoptosis by inhibition of the phosphatidylinositol-3 kinase/protein kinase B pathway and showed neuroprotective properties against neurotoxicity caused by the β-amyloid peptide in hippocampal slices [68]. The lack of AR effect on N2a culture might be attributed to differences in cellular mechanisms from CTX culture, mediating OA-induced tau phosphorylation at multiple levels and different sites.

LiCl is well-known to inhibit GSK3 and other kinases [108]. In CTX culture, LiCl caused dramatic inhibition of basal and OA-induced tau hyperphosphorylation at all tested tau epitopes. Consistent with previous reports, LiCl was shown to reduce tau phosphorylation in cultured cells, Ex-vivo rat brain slices, and rat brains at different AD-related tau epitopes [43, 108, 128–131]. Unexpectedly, LiCl showed an opposite impact on N2a culture by increasing OA-induced tau hyperphosphorylation and oligomerization at multiple tested tau epitopes. To the best of our knowledge, this effect is reported for the first time in cell culture. However, there are biological targets for LiCl that might have resulted in an adverse event. For instance, one hypothesis states that LiCl is a competitive inhibitor of GSK-3 to Mg^2+^, but not competitive to the substrate or ATP. Another theory proposes that LiCl causes potassium deprivation [132].

The use of CDK5 inhibitor Roscovitine in CTX culture substantially reduced basal and OA-induced tau hyperphosphorylation at CP13 (pSer202), RZ3 (pT231, PHF-1(pSer396/pSer404) and AT270 (pThr181). Roscovitine reduced basal phosphorylation at AT8 (pSer202/pThr205) but did not affect the OA-induced tau hyperphosphorylation, reflecting its specificity and the selectivity to our cell models. Similarly, several recent studies revealed that inhibiting CDK5 with Roscovitine had neuroprotective properties against neurodegenerative conditions caused by decreasing tau phosphorylation [75, 108, 133]. Like LiCl, Roscovitine resulted in opposite effects in the N2a cells by increasing phosphorylation at CP13 (pSer202) and PHF-1 (pSer396/pSer404).

Another protein kinase that has recently received consideration as a pharmaceutical target is the tyrosine kinase Fyn, which has been linked with the amyloid pathway and tau phosphorylation through the N-terminal domain in dendrites [24]. Saracatinib (also known as AZD0530) is a small molecular inhibitor that has high potency for Src and Fyn kinases [24, 27–29]. Fyn can physically associate with tau and phosphorylate residues by interacting through its SH3 domain with SH3-binding domains in tau **(Fig 6)** [134]. In our experiments, Saracatinib reduced both basal and OA-induced tau hyperphosphorylation (67 kDa) in N2a and CTX primary cultures at the epitopes: CP13 (pSer202), RZ3 (pThr231), PHF-1 (pSer396/pSer404) and AT270 (pThr181). Saracatinib did not affect the pSer202/pThr205 (AT8) site, suggesting that Fyn does not phosphorylate Thr205 residue in our experimental tauopathy model.

Cyclosporine (CsA) or FK506 is an 11 amino acid cyclic non-ribosomal peptide used as an immunosuppressant. CsA induces neuroprotective properties through inhibiting specifically enzyme activity by binding to cyclophilin, forming a complex that inhibits calcineurin (PP3) [135] **(Fig 6, Table 2)**. Several findings have shown that calcineurin inhibition increases tau hyperphosphorylation, and cells treated with CsA could induce the process [136, 137]. In the present study, it was found that treatment with CsA alone did not result in any significant increase in tau levels or tau phosphorylation, which lies in agreement with a study done similarly [138], and reported complete inhibition of OA-induced tau hyperphosphorylation and oligomerization in N2a cells at the examined tau epitopes. In CTX culture, CsA produced a lower but still considerable reduction of OA-induced tau phosphorylation compared to N2a neuronal culture. These data suggest that PP2A is the main enzyme that regulates tau dephosphorylation in our culture system rather than PP3 at the tested sites. Moreover, we propose that CsA inhibits PP3 by blocking its binding to the calcium-dependent calmodulin, required for CaMKII to be active, thereby decreasing tau hyperphosphorylation **(Fig 6, Table 2)**.

### Limitation and future directions

Some limitations to the study include the idea that these compound dilutions are arbitrary or based on references using different cell systems. Treatment is often in the high micromolar range, where compounds act on more than their primary target. Using serial dilution of the inhibitors and the generation of an EC_50_ in clinical samples will provide insights on the correct dosage. Additional techniques can be used, such as ELISA, which aid its potential use in higher throughput screens. These systems are highly specific, but a wider variety of auto-antibody assays can also provide more confidence in the efficacy of some inhibitors. The discrepancy in response between the N2a cell line and CTX also cautions the interpretation of the screen. Having a wider variety of samples from distinct parts of the brain might give more precise evidence toward the study.

## Conclusions

In this study, OA was used to induce a cell model of tauopathy in neuroblastoma and differentiated neuronal culture and screen for various pharmaceutical drug candidates. We provided a side-by-side comparison of drug candidates that are well described regarding tauopathies such as Alzheimer’s (Saracatinib, LiCl, AR) and other prospects that have been minimally studied in application to potential therapies (TBB and CsA). TBB and CsA warrant further test design involving an animal model of tauopathy. Exploration of agents that inhibit tauopathy progression is important as recent studies implicate pre-fibrillar hyperphosphorylated tau as the toxic species in AD, CTE, and other neurodegenerative diseases, therefore, re-establishing the interest in tau kinase inhibitors development at putative neurotherapies, which could translate into human clinical trials.

## Supporting information

S1 Fig Effect of cyclosporin A on OA-induced tau hyperphosphorylation in mouse N2a cells. The same experimental design mentioned in **Fig 2** was used to test CsA in N2a cell culture. Twenty micrograms of protein extract were used for the analysis of tau. Calpain and caspase-3 inhibitors (S+Z) were added to all experimental conditions, including the control samples. CsA is known to inhibit the phosphatase activity of calcineurin (PP3). In the presented experiment, it is used to assess its kinase inhibition potential on the monomeric and oligomeric p-tau induced by OA. (a). Immunoblots of N2a neuronal culture protein extracts showing antibodies directed against major tau phosphorylation sites. Two additional p-tau antibodies were used (AT270 and RZ3) to assess the phosphorylation sites at pThr181 and pThr231, respectively. RZ3 and AT270 detected distinctive monomeric p-tau bands at 48 kDa, and 55 kDa, respectively. Total tau levels were probed using DA9 (a.a. 102–145) in N2a cells. Blue colored labels correspond to monomeric or oligomeric p-tau species. Immunoblots were probed with αII-spectrin antibody to monitor calpain and caspase-3 mediated proteolysis. (b). Immunoblots quantification of N2a. The ratio of phosphorylation epitopes levels over β-actin levels ± SD are represented as a percentage of control. n = 3 per condition. For multiple comparisons, one-way ANOVA followed by the Bonferroni’s post-hoc test was performed. *p<0.05, **p<0.01, ***p<0.001, ****p<0.0001, ns: non-significant.
(PDF)

S2 Fig Effect of additional two GSK-3 protein kinase inhibitors on OA-induced tau hyperphosphorylation and oligomerization in N2a cells (with cell-death linked protease inhibitors (calpain/caspase inhibitors). A continuation of Fig 2 experiments is presented to include two other potent GSK-3 kinase inhibitors, AR and A-107. The specific experimental treatments are as described in materials and methods. (a). Immunoblots of N2a cells extracted protein using p-tau antibodies (CP13 and PHF-1), total tau (DA9), and αII-Spectrin. αII-Spectrin was probed to assess cell apoptosis monitored SBDP150/145 kDa and SBDP120 kDa. Kinase inhibition of phosphorylation and oligomerization was monitored by evaluating the levels of p-tau antibodies and total tau (blue arrows) and non-phospho tau (black arrows). For all conditions, S+Z were added for 1h before the treatments. (b). Immunoblots quantification and statistical analysis. All data are normalized to β-actin and are expressed as a percentage of control. Data are presented as ± SEM for n = 3. Statistical analysis was performed with one-way ANOVA. For multiple comparisons, one-way ANOVA followed by Bonferroni’s post-hoc test was performed. *p<0.05, **p<0.01, ***p<0.001, ****p<0.0001 and ns: non-significant.
(PDF)

S3 Fig Cyclosporin A inhibits physiological and OA-induced Tau hyperphosphorylation in rat primary cerebrocortical neuronal culture. The experimental procedures were followed, as described in **Fig 4** and **Fig 5** legends. Primary neuronal cultures (CTX) were fully differentiated and had healthy neurites when examined under the microscope. All wells were pretreated with S+Z for 1h. For conditions that did not include OA, cultures were treated with CsA for 6h. For OA-induced conditions, OA was added for 24h followed by CsA for 6h. A reverse-time course was followed, and all experimental conditions were collected and analyzed at the same time. Twenty micrograms of CTX culture extracts were run on SDS-PAGE, followed by western blotting. (a). Immunoblots of CTX culture protein extracts. CTX culture using antibodies directed against major tau phosphorylation sites including: CP13 (pSer202), PHF-1 (pSer396/pSer404), RZ3 (pThr231), AT8 (pSer202/pThr205), AT270 (pThr181). Total tau levels were probed using DA31 (a.a. 102–145). The 67 kDa assigned as a monomeric p-tau band, and the 63 kDa band was assigned as monomeric non-phospho tau at the different studied epitopes. (b). Immunoblots quantification. The ratio of phosphorylation epitopes levels over β-actin levels ± SD are represented as a percentage of control. n = 3 per condition. For multiple comparisons, one-way ANOVA followed by Bonferroni’s post-hoc test was performed. *p<0.05, **p<0.01, ***p<0.001, ****p<0.0001, ns: non-significant.
(PDF)

S1 Raw Images Images were captured with the computer-assisted densitometric *scanning* (*Epson* 8836XL high-resolution *scanner*) and NIH Image J densitometry software. MW, GE Healthcare rainbow full range molecular weight marker.
(PDF)

## List of abbreviations

(A-1070722): 1-(7-methoxyquinolin-4-yl)-3-(6-(trifluoromethyl) pyridin-2-yl
AD: Alzheimer’s disease
AR-A014418: N-(4-methoxybenzyl)-N'-(5-nitro-1,3-thiazol-2-yl) urea
AZD0530: Saracatinib
BCIT/NBT: 5-bromo-4-chloro- 3-indolyl phosphate
CaMKII: Ca^2+^/calmodulin-dependent protein kinase II
CDK5: Cyclin-dependent kinase 5
CK2: Casein kinase
CKII: Casein kinase II
CsA: Cyclosporin A
CTE: Chronic traumatic encephalopathy
CTX: Neuronal cortical cell cultures
DTT: Dithiothreito
DYRK1A/2: Dual-specificity tyrosine kinase phosphorylation and regulated kinase-1A/2
EGTA: Ethylene glycol-bis (β-aminoethyl ether)-N,N,N',N'-tetraacetic acid
GSK-3: Glycogen synthase kinase 3 inhibitors
HMW: High Molecular Weight
K252a: Staurosporine analog, non-selective cell permeable Protein Kinase Inhibitor; (9S,10R,12R)-2,3,9,10,11,12-Hexahydro-10-hydroxy-9-methyl-1-oxo-9,12-epoxy-1H-diindolo[1,2,3-fg:3',2',1'-kl]pyrrolo[3,4-i][1,6]benzodiazocine-10-carboxylic acid methyl ester
LiCl: Lithium Chloride
LMW-MT: Low molecular weight monomeric tau
MLCK: Myosin light-chain kinase
N2A: Neuroblastoma cell line
NFT: Neurofibrillary tangles
NMDAR: N-methyl-D-aspartate receptors
OA: Okadaic acid
PP1: Protein Phosphatase 1
PP2A: Protein Phosphatase 2A
Roscovitine: (2R)-2-1-butanol
Saracatinib: N-(5-chloro-1,3-benzodioxol-4-yl)-7-[2-(4-methylpiperazin-1-yl)ethoxy]-5-(tetrahydro-2H-pyran-4-yloxy)quinazolin-4-amine
SBDP: Spectrin breakdown products
SNJ-1945: Amphipathic ketoamide
STS: Staurosporine; (5S,6R,7R,9R)-6-methoxy-5-methyl-7-(methylamino)-6,7,8,9,15,16-hexahydro-17-oxa-4b,9a,15-triaza-5,9-methanodibenzo[b,h]cyclonona[jkl]cyclopenta[e]-as-indacen-14(5h)-one
TBB: 4,5,6,7-tetrabromobenzotriazole
Z-DCB: Caspase-3; Z-Asp-2,6-Dichlorobenzoyloxymethyl Ketone
