# Supplementary material for: Screening of tau protein kinase inhibitors in a tauopathy-relevant cell-based model of tau hyperphosphorylation and oligomerization
Source: PLoS One. 2020 Jul 21;15(7):e0224952. doi: 10.1371/journal.pone.0224952 (PMC7373298; doi:10.1371/journal.pone.0224952)
Supplement: S1 Fig — The same experimental design mentioned in Fig 2 was used to test CsA in N2a cell culture. Twenty micrograms of protein extract were used for the analysis of tau. Calpain and caspase-3 inhibitors (S+Z) were added to all experimental conditions, including the control samples. CsA is known to inhibit the phosphatase activity of calcineurin (PP3). In the presented experiment, it is used to assess its kinase inhibition potential on the monomeric and oligomeric p-tau induced by OA. (a). Immunoblots of N2a neuronal culture protein extracts showing antibodies directed against major tau phosphorylation sites. Two additional p-tau antibodies were used (AT270 and RZ3) to assess the phosphorylation sites at pThr181 and pThr231, respectively. RZ3 and AT270 detected distinctive monomeric p-tau bands at 48 kDa, and 55 kDa, respectively. Total tau levels were probed using DA9 (a.a. 102–145) in N2a cells. Blue colored labels correspond to monomeric or oligomeric p-tau species. Immunoblots were probed with αII-spectrin antibody to monitor calpain and caspase-3 mediated proteolysis. (b). Immunoblots quantification of N2a. The ratio of phosphorylation epitopes levels over β-actin levels ± SD are represented as a percentage of control. n = 3 per condition. For multiple comparisons, one-way ANOVA followed by the Bonferroni’s post-hoc test was performed. *p<0.05, **p<0.01, ***p<0.001, ****p<0.0001, ns: non-significant. (PDF) [file pone.0224952.s001.pdf]

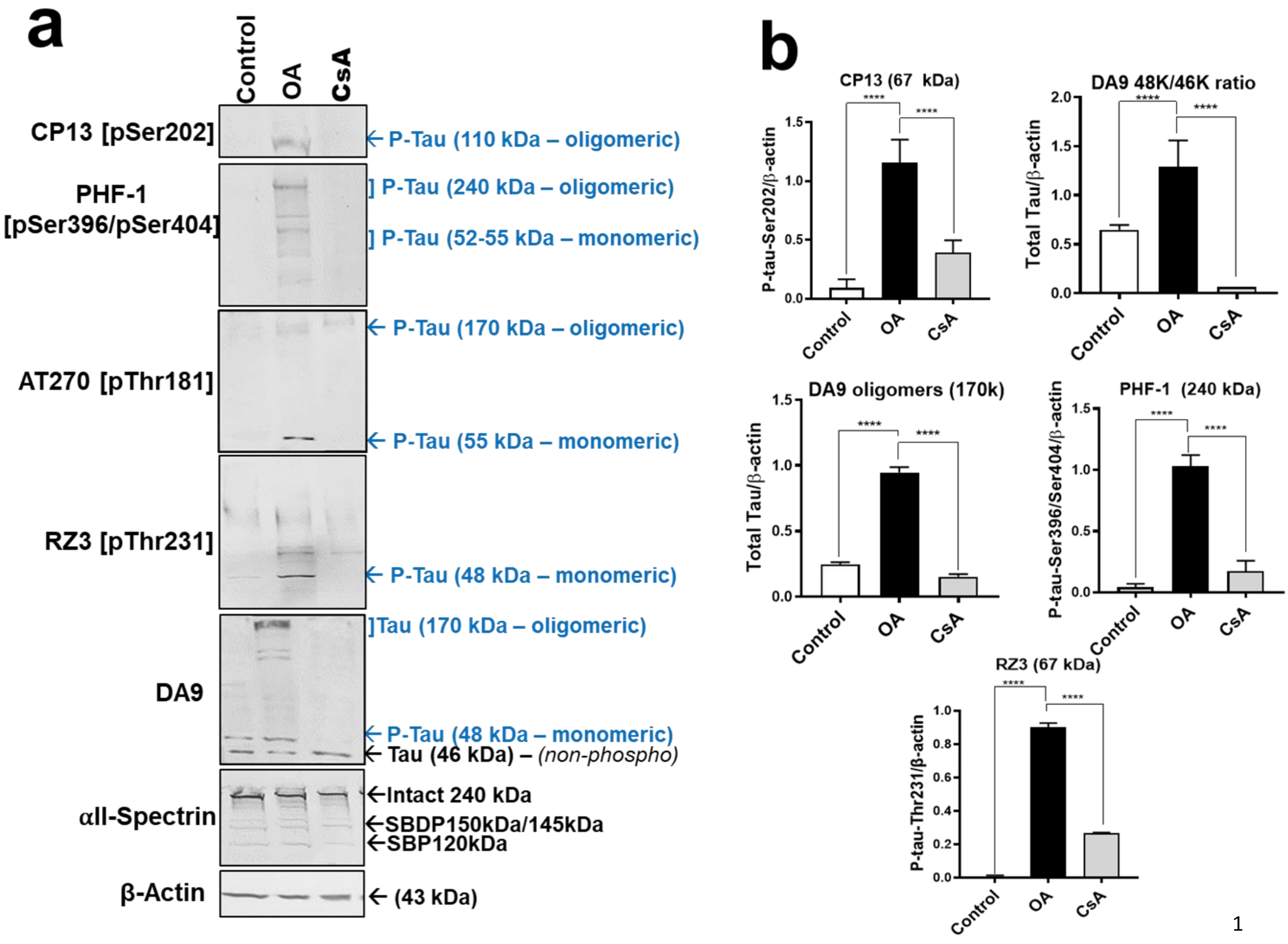
a
Control
OA
CsA
CP13 [pSer202]
PHF-1
[pSer396/pSer404]
AT270 [pThr181]
RZ3 [pThr231]
DA9
αII-Spectrin
β-Actin
← P-Tau (110 kDa – oligomeric)
] P-Tau (240 kDa – oligomeric)
] P-Tau (52-55 kDa – monomeric)
← P-Tau (170 kDa – oligomeric)
← P-Tau (55 kDa – monomeric)
← P-Tau (48 kDa – monomeric)
]Tau (170 kDa – oligomeric)
← P-Tau (48 kDa – monomeric)
← Tau (46 kDa) – (non-phospho)
←Intact 240 kDa
←SBDP150kDa/145kDa
←SBP120kDa
← (43 kDa)
b
CP13 (67 kDa)
P-tau-Ser202/β-actin
DA9 48K/46K ratio
Total Tau/β-actin
DA9 oligomers (170k)
Total Tau/β-actin
PHF-1 (240 kDa)
P-tau-Ser396/Ser404/β-actin
RZ3 (67 kDa)
P-tau-Thr231/β-actin
****
Control
OA
CsA

S1 Fig. Effect of cyclosporin A on OA-induced tau hyperphosphorylation in mouse N2a cells. The same experimental design mentioned in Fig2 was used to test CsA in N2a cell culture. Twenty micrograms of protein extract were used for the analysis of tau. Calpain and caspase-3 inhibitors (S+Z) were added to all experimental conditions, including the control samples. CsA inhibits the phosphatase activity of calcineurin (PP3). In the presented experiment, it is used to assess its kinase inhibition potential on the monomeric and oligomeric p-tau induced by OA. (a). Immunoblots of N2a cells protein extracts showing antibodies directed against major tau phosphorylation sites. Two additional p-tau antibodies were used (AT270 and RZ3) to assess the phosphorylation sites at pThr181 and pThr231, respectively. RZ3 and AT270 detected distinctive monomeric p-tau bands at 48 kDa, and 55 kDa, respectively. Total tau levels were probed using DA9 (a.a. 102-145) in N2a cells. Blue colored labels correspond to monomeric or oligomeric p-tau species. Immunoblots were probed with αII-spectrin antibody to monitor calpain and caspase-3 mediated proteolysis. (b). Immunoblots quantification of N2a.The ratio of phosphorylation epitopes levels over β-actin levels ± SD are represented as a percentage of control. n=3 per condition. For multiple comparisons, one-way ANOVA followed by the Bonferroni's post-hoc test was performed. *p<0.05, **p<0.01, ***p<0.001, ****p<0.0001, ns: non-significant.
