# Supplementary material for: Screening of tau protein kinase inhibitors in a tauopathy-relevant cell-based model of tau hyperphosphorylation and oligomerization
Source: PLoS One. 2020 Jul 21;15(7):e0224952. doi: 10.1371/journal.pone.0224952 (PMC7373298; doi:10.1371/journal.pone.0224952)
Supplement: S2 Fig — A continuation of Fig 2 experiments is presented to include two other potent GSK-3 kinase inhibitors, AR and A-107. The specific experimental treatments are as described in materials and methods. (a). Immunoblots of N2a cells extracted protein using p-tau antibodies (CP13 and PHF-1), total tau (DA9), and αII-Spectrin. αII-Spectrin was probed to assess cell apoptosis monitored SBDP150/145 kDa and SBDP120 kDa. Kinase inhibition of phosphorylation and oligomerization was monitored by evaluating the levels of p-tau antibodies and total tau (blue arrows) and non-phospho tau (black arrows). For all conditions, S+Z were added for 1h before the treatments. (b). Immunoblots quantification and statistical analysis. All data are normalized to β-actin and are expressed as a percentage of control. Data are presented as ± SEM for n = 3. Statistical analysis was performed with one-way ANOVA. For multiple comparisons, one-way ANOVA followed by Bonferroni’s post-hoc test was performed. *p<0.05, **p<0.01, ***p<0.001, ****p<0.0001 and ns: non-significant. (PDF) [file pone.0224952.s002.pdf]

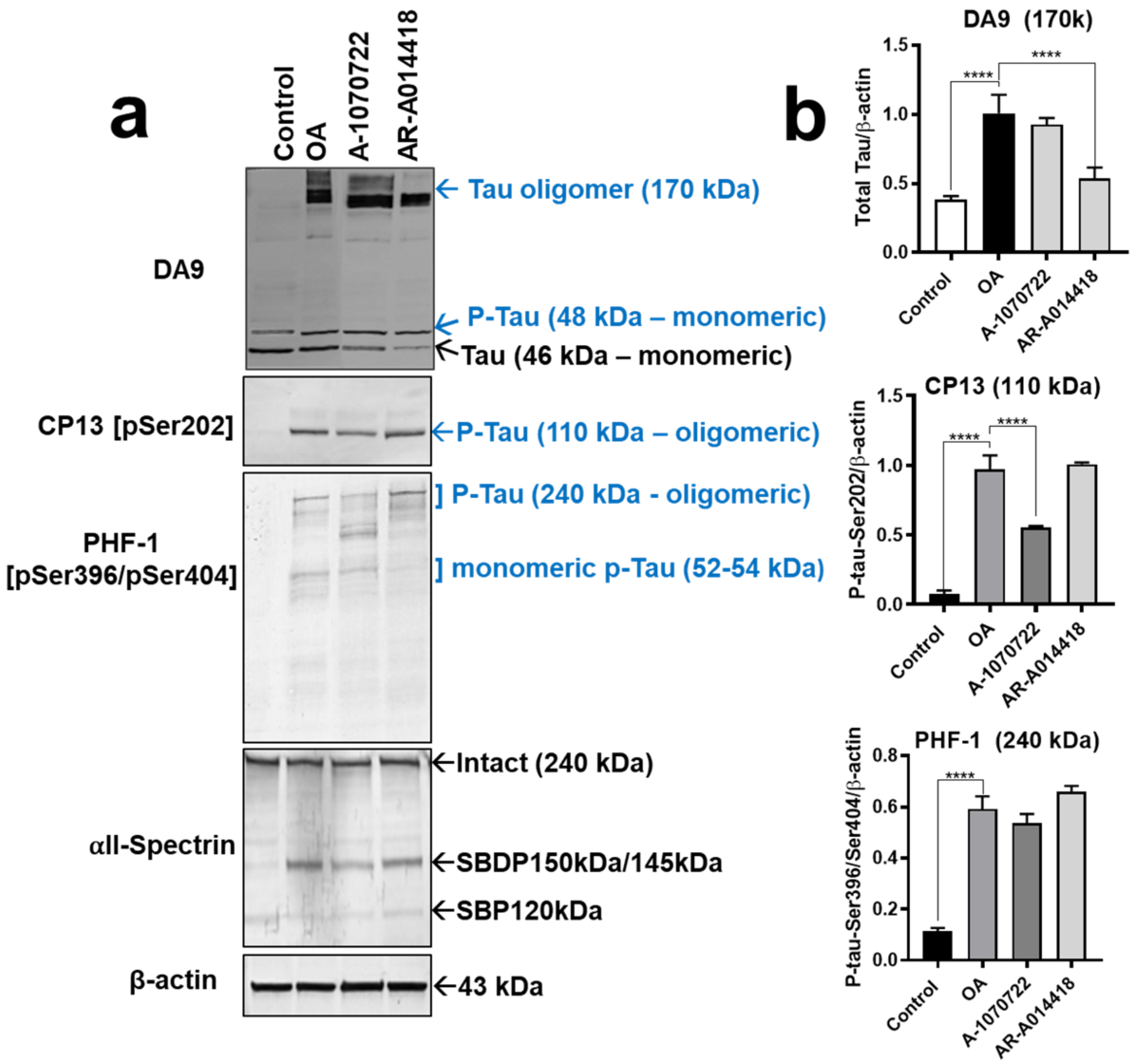
a
Control
OA
A-1070722
AR-A014418
DA9
← Tau oligomer (170 kDa)
← P-Tau (48 kDa – monomeric)
← Tau (46 kDa – monomeric)
CP13 [pSer202]
← P-Tau (110 kDa – oligomeric)
PHF-1
[pSer396/pSer404]
] P-Tau (240 kDa - oligomeric)
] monomeric p-Tau (52-54 kDa)
αII-Spectrin
← Intact (240 kDa)
← SBDP150kDa/145kDa
← SBP120kDa
β-actin
← 43 kDa
b
DA9 (170k)
Total Tau/β-actin
****
****
CP13 (110 kDa)
P-tau-Ser202/β-actin
****
****
PHF-1 (240 kDa)
P-tau-Ser396/Ser404/β-actin
****

S2 Fig. Effect of additional two GSK-3 protein kinase inhibitors on OA-induced tau hyperphosphorylation and oligomerization in N2a cells (with cell-death linked protease inhibitors (calpain/caspase inhibitors). A continuation of Fig2 experiment is presented to include two other potent GSK-3 kinase inhibitors, AR and A-107. The specific experimental treatments are as described in materials and methods. (a). Immunoblots of N2a cells extracted protein using p-tau antibodies (CP13 and PHF-1), total tau (DA9), and αII-Spectrin. αII-Spectrin was probed to assess cell apoptosis monitored SBDP150/145 kDa and SBDP120 kDa. Kinase inhibition of phosphorylation and oligomerization was monitored by evaluating the levels of p-tau antibodies and total tau (blue arrows) and non-phospho tau (black arrows). For all conditions, S+Z were added for 1h before the treatments. (b). Immunoblot quantifications and statistical analysis. All data are normalized to β-actin and are expressed as a percentage of control. Data are presented as ± SEM for n=3. Statistical analysis was performed with one-way ANOVA. For multiple comparisons, one-way ANOVA followed by the Bonferroni's post-hoc test was performed. *$p<0.05$, **$p<0.01$, ***$p<0.001$, ****$p<0.0001$ and ns: non-significant.
