# Supplementary material for: Screening of tau protein kinase inhibitors in a tauopathy-relevant cell-based model of tau hyperphosphorylation and oligomerization
Source: PLoS One. 2020 Jul 21;15(7):e0224952. doi: 10.1371/journal.pone.0224952 (PMC7373298; doi:10.1371/journal.pone.0224952)
Supplement: S3 Fig — The experimental procedures were followed, as described in Fig 4 and Fig 5 legends. Primary neuronal cultures (CTX) were fully differentiated and had healthy neurites when examined under the microscope. All wells were pretreated with S+Z for 1h. For conditions that did not include OA, cultures were treated with CsA for 6h. For OA-induced conditions, OA was added for 24h followed by CsA for 6h. A reverse-time course was followed, and all experimental conditions were collected and analyzed at the same time. Twenty micrograms of CTX culture extracts were run on SDS-PAGE, followed by western blotting. (a). Immunoblots of CTX culture protein extracts. CTX culture using antibodies directed against major tau phosphorylation sites including: CP13 (pSer202), PHF-1 (pSer396/pSer404), RZ3 (pThr231), AT8 (pSer202/pThr205), AT270 (pThr181). Total tau levels were probed using DA31 (a.a. 102–145). The 67 kDa assigned as a monomeric p-tau band, and the 63 kDa band was assigned as monomeric non-phospho tau at the different studied epitopes. (b). Immunoblots quantification. The ratio of phosphorylation epitopes levels over β-actin levels ± SD are represented as a percentage of control. n = 3 per condition. For multiple comparisons, one-way ANOVA followed by Bonferroni’s post-hoc test was performed. *p<0.05, **p<0.01, ***p<0.001, ****p<0.0001, ns: non-significant. (PDF) [file pone.0224952.s003.pdf]

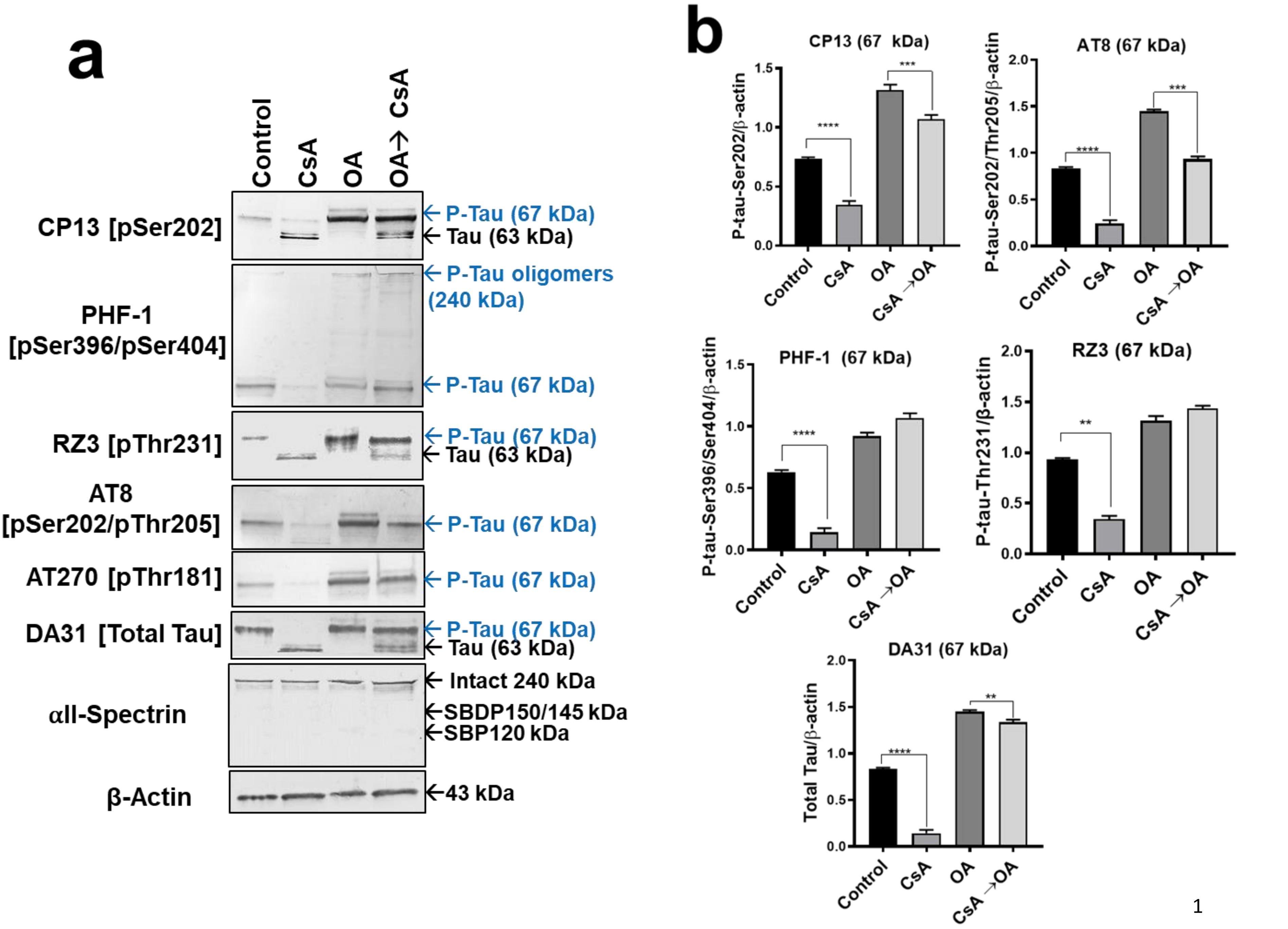
a
Control
CsA
OA
OA→ CsA
CP13 [pSer202]
PHF-1
[pSer396/pSer404]
RZ3 [pThr231]
AT8
[pSer202/pThr205]
AT270 [pThr181]
DA31 [Total Tau]
αII-Spectrin
β-Actin
← P-Tau (67 kDa)
← Tau (63 kDa)
← P-Tau oligomers
(240 kDa)
← P-Tau (67 kDa)
← P-Tau (67 kDa)
← Tau (63 kDa)
← P-Tau (67 kDa)
← P-Tau (67 kDa)
← P-Tau (67 kDa)
← Tau (63 kDa)
← Intact 240 kDa
←SBDP150/145 kDa
←SBP120 kDa
←43 kDa
b
CP13 (67 kDa)
P-tau-Ser202/β-actin
AT8 (67 kDa)
P-tau-Ser202/Thr205/β-actin
PHF-1 (67 kDa)
P-tau-Ser396/Ser404/β-actin
RZ3 (67 kDa)
P-tau-Thr231/β-actin
DA31 (67 kDa)
Total Tau/β-actin
Control
CsA
OA
CsA →OA

S3 Fig. Cyclosporin A inhibits physiological and OA-induced Tau hyperphosphorylation in rat primary cerebrocortical neuronal culture. The experimental procedures were followed as described in Fig4 and Fig5. Primary neuronal cultures (CTX) were fully differentiated and had healthy neurites when examined under the microscope. All wells were pretreated with S+Z for 1h. For conditions that did not include OA, cultures were treated with CsA for 6h. For OA-induced conditions, OA was added for 24h followed by CsA for 6h. A reverse-time course was followed, and all experimental conditions were collected and analyzed at the same time. Twenty micrograms of CTX culture extract were run on SDS-PAGE followed by western blotting. **(a)**. Immunoblots of CTX culture protein extracts. CTX culture using antibodies directed against major tau phosphorylation sites including: CP13 (pSer202), PHF-1 (pSer396/pSer404), RZ3 (pThr231), AT8 (pSer202/pThr205), AT270 (pThr181). Total tau levels were probed using DA31 (a.a. 102-145). The 67 kDa assigned as monomeric p-tau band, and the 63 kDa band was assigned as monomeric non-phospho tau at the different studied epitopes. **(c)**. Immunoblots quantification. The ratio of phosphorylation epitopes levels over β-actin levels ± SD are represented as a percentage of control. n=3 per condition. For multiple comparisons, one-way ANOVA followed by the Bonferroni's post-hoc test was performed. *$p<0.05$, **$p<0.01$, ***$p<0.001$, ****$p<0.0001$, ns: non-significant.
