# Supplementary material for: Screening of tau protein kinase inhibitors in a tauopathy-relevant cell-based model of tau hyperphosphorylation and oligomerization
Source: PLoS One. 2020 Jul 21;15(7):e0224952. doi: 10.1371/journal.pone.0224952 (PMC7373298; doi:10.1371/journal.pone.0224952)
Supplement: S1 Raw Images — MW, GE Healthcare rainbow full range molecular weight marker. (PDF) [file pone.0224952.s004.pdf]

Raw image for the production of S1 Fig a. Images were captured with the computer-assisted densitometric *scanning* (*Epson* 8836XL high-resolution *scanner* and NIH Image J densitometry software. MW, GE Healthcare rainbow full range molecular weight marker.

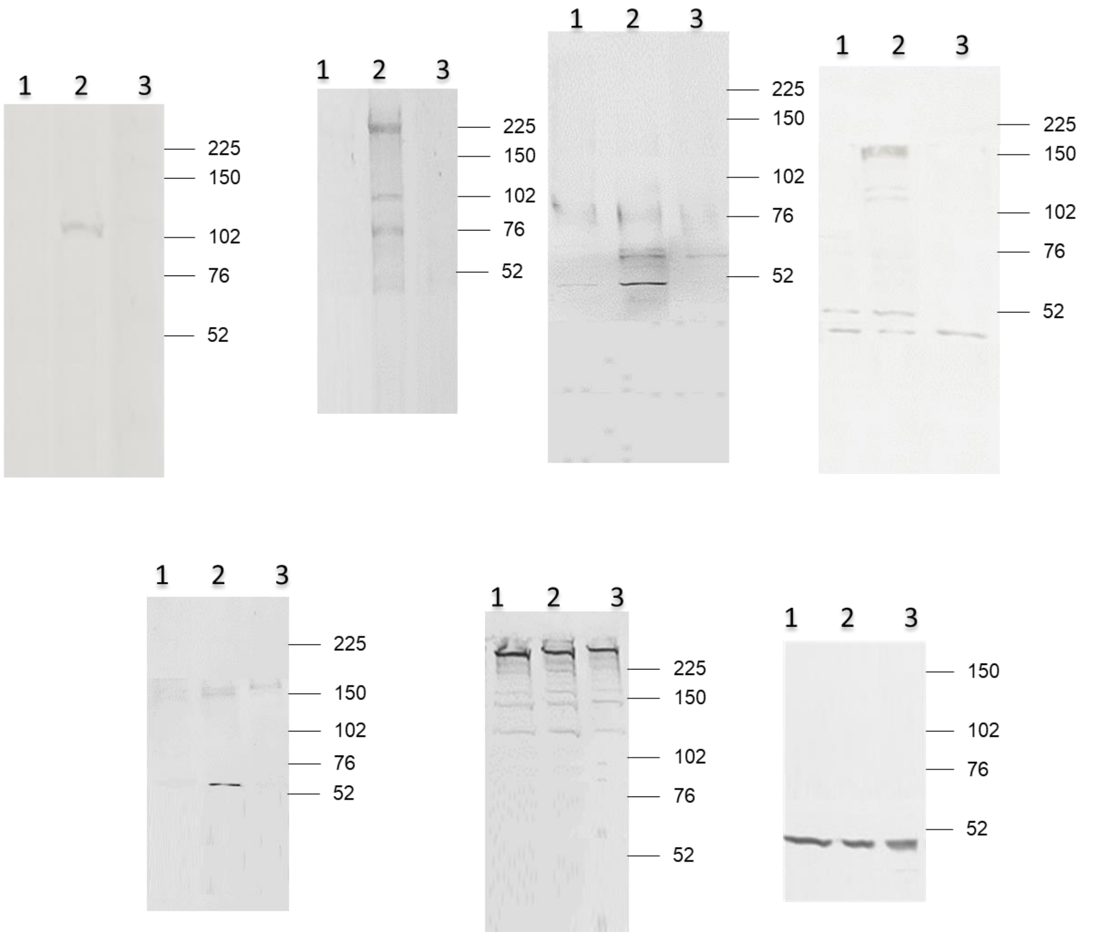

Raw image for the production of S2 Fig a. Images were captured with the computer-assisted densitometric *scanning* (*Epson* 8836XL high-resolution *scanner* and NIH Image J densitometry software. MW, GE Healthcare rainbow full range molecular weight marker.

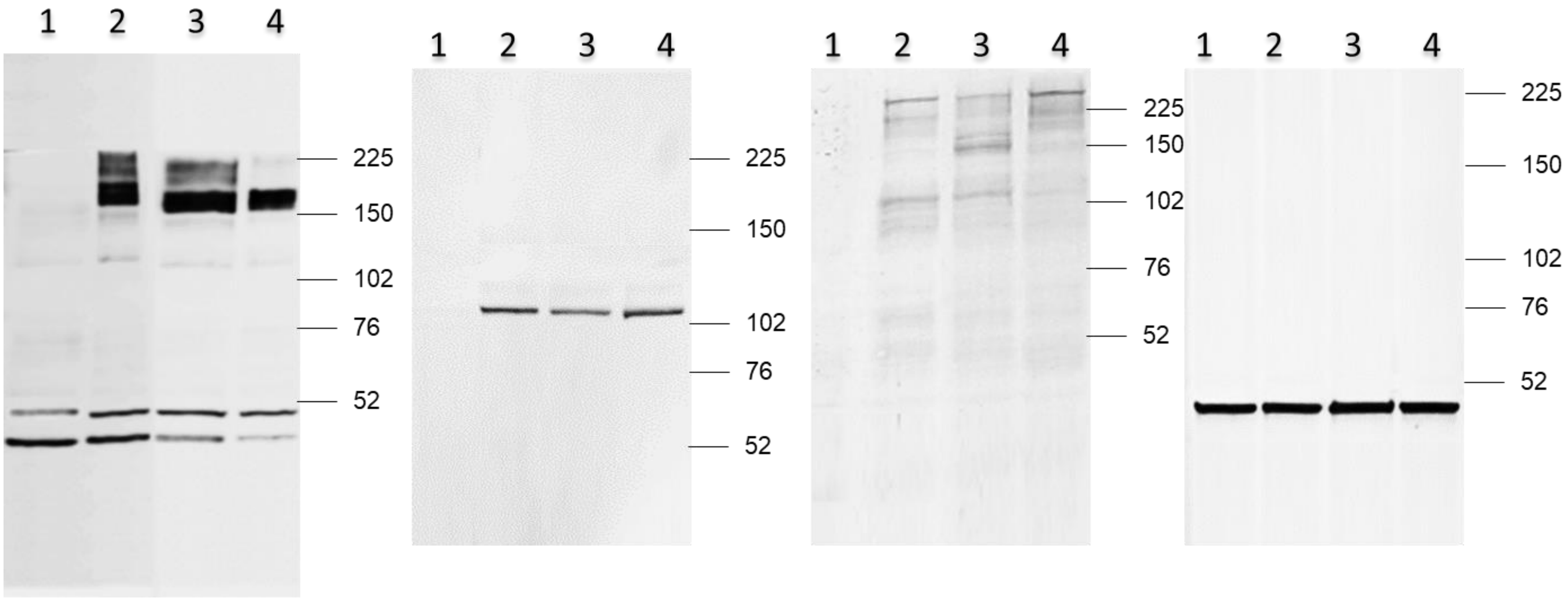

Raw image for the production of S3 Fig a. Images were captured with the computer-assisted densitometric *scanning* (*Epson* 8836XL high-resolution *scanner* and NIH Image J densitometry software. MW, GE Healthcare rainbow full range molecular weight marker.

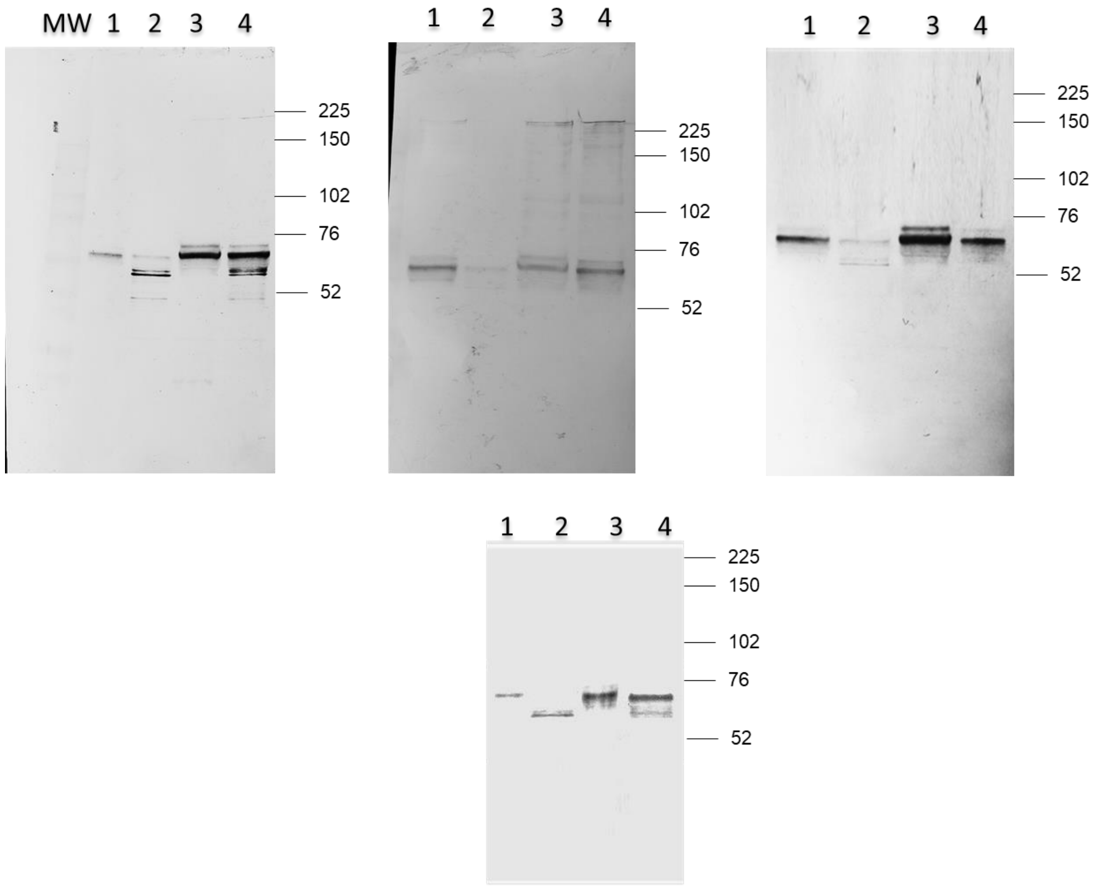

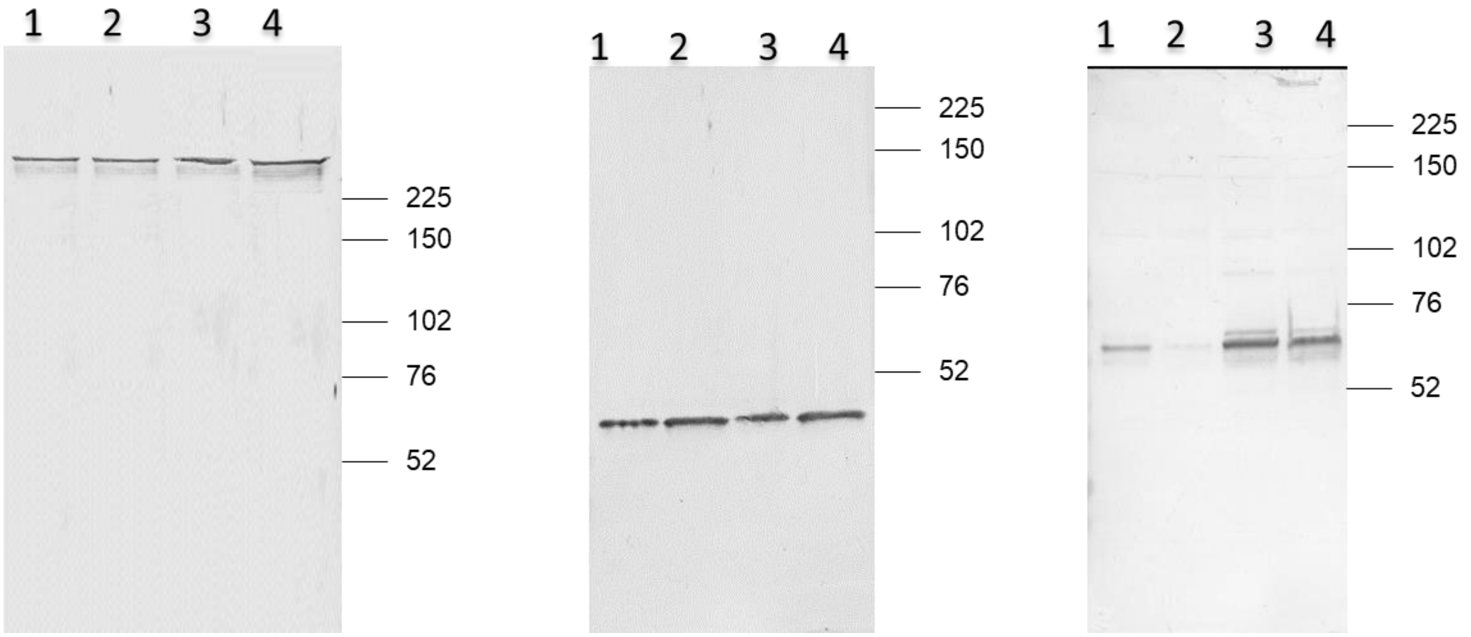
1 2 3 4
225
150
102
76
52
1 2 3 4
225
150
102
76
52
1 2 3 4
225
150
102
76
52

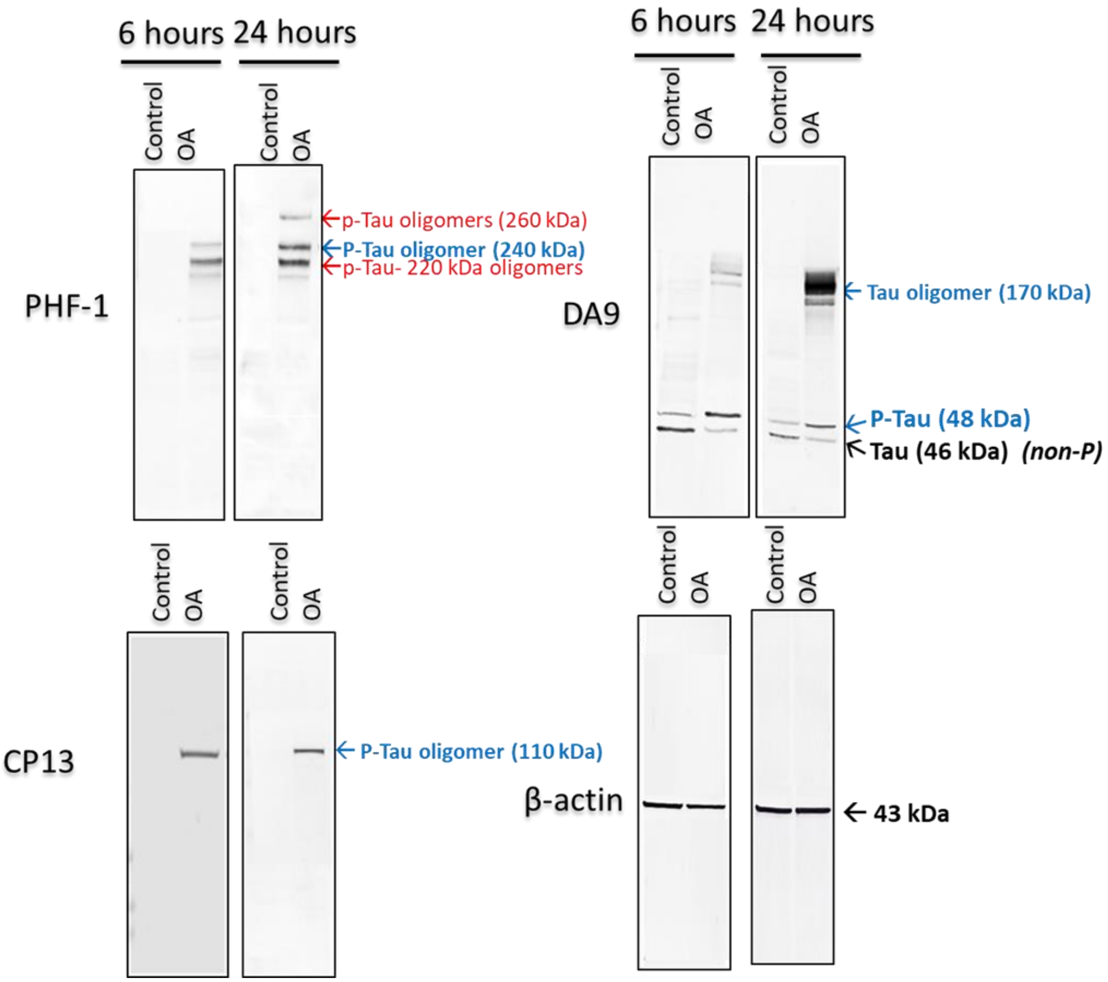

Raw image for the production of Fig 1a. Images were captured with the computer-assisted densitometric *scanning* (*Epson* 8836XL high-resolution *scanner* and NIH Image J densitometry software. MW, GE Healthcare rainbow full range molecular weight marker.

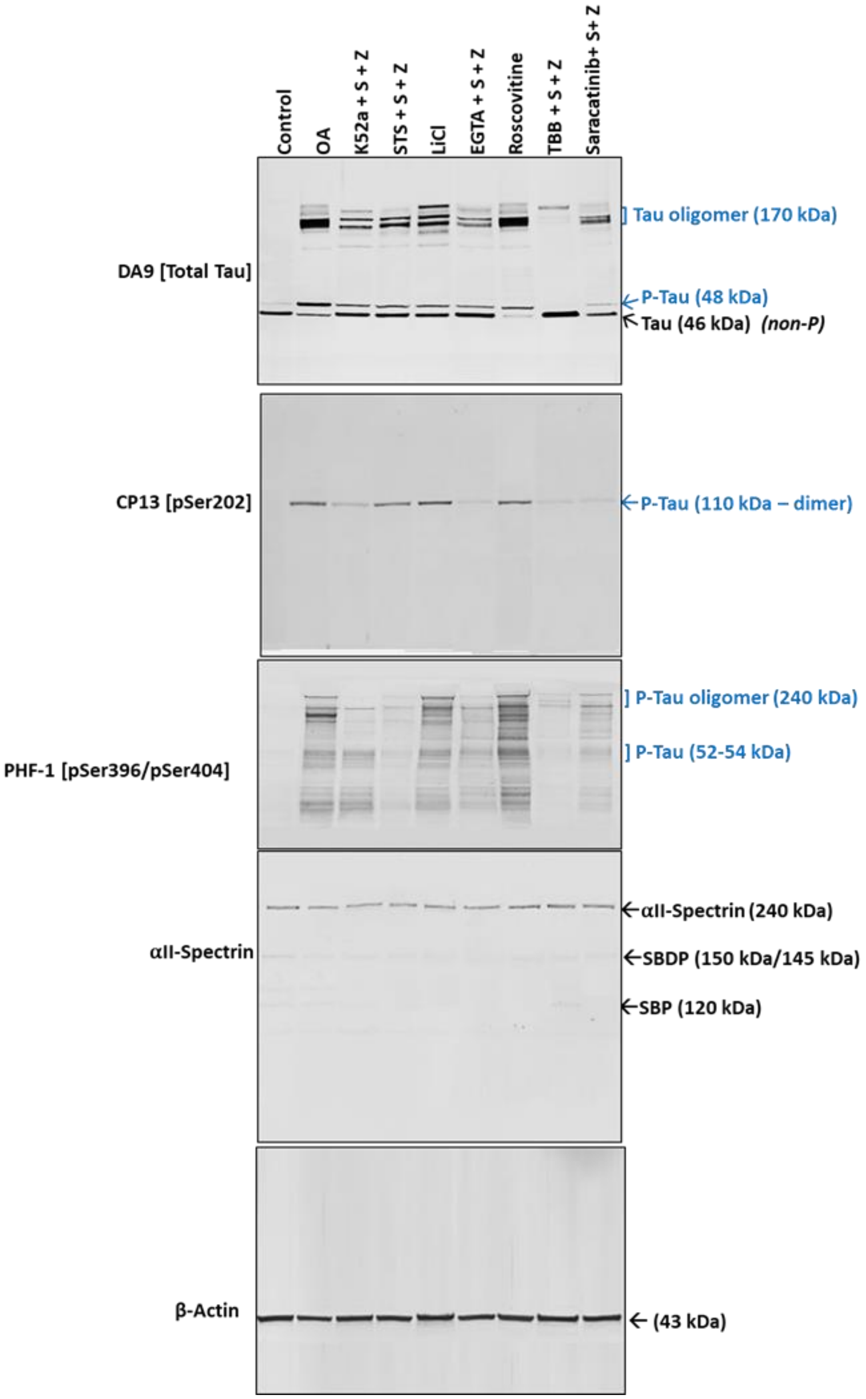

Raw image for the production of Fig 2a. Images were captured with the computer-assisted densitometric *scanning* (*Epson* 8836XL high-resolution *scanner* and NIH Image J densitometry software. MW, GE Healthcare rainbow full range molecular weight marker.

weight marker.

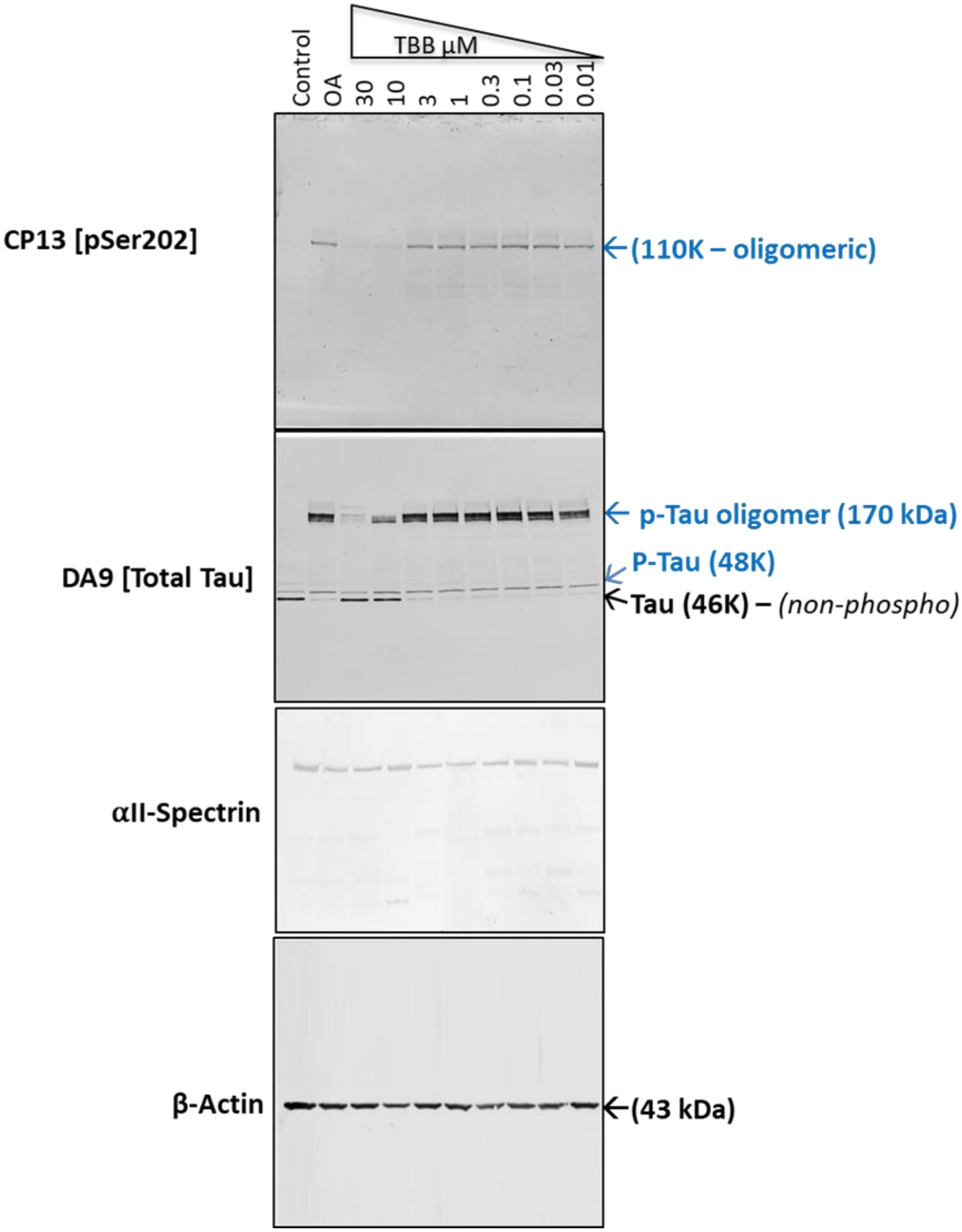

Raw image for the production of Fig 3a. Images were captured with the computer-assisted densitometric *scanning* (*Epson* 8836XL high-resolution *scanner* and NIH Image J densitometry software. MW, GE Healthcare rainbow full range molecular weight marker.

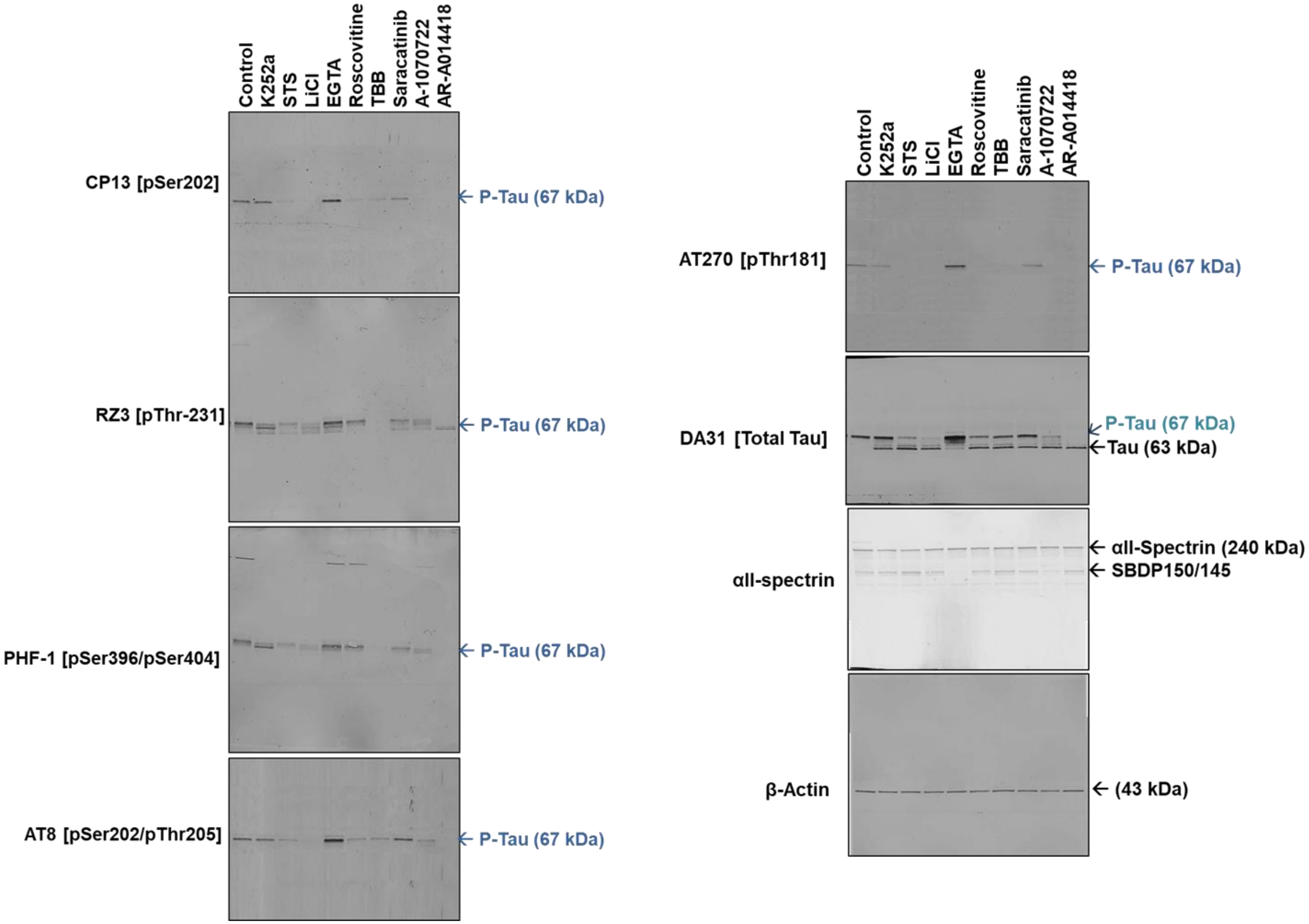

Raw image for the production of Fig 4a. Images were captured with the computer-assisted densitometric *scanning* (*Epson* 8836XL high-resolution *scanner* and NIH Image J densitometry software. MW, GE Healthcare rainbow full range molecular weight marker.

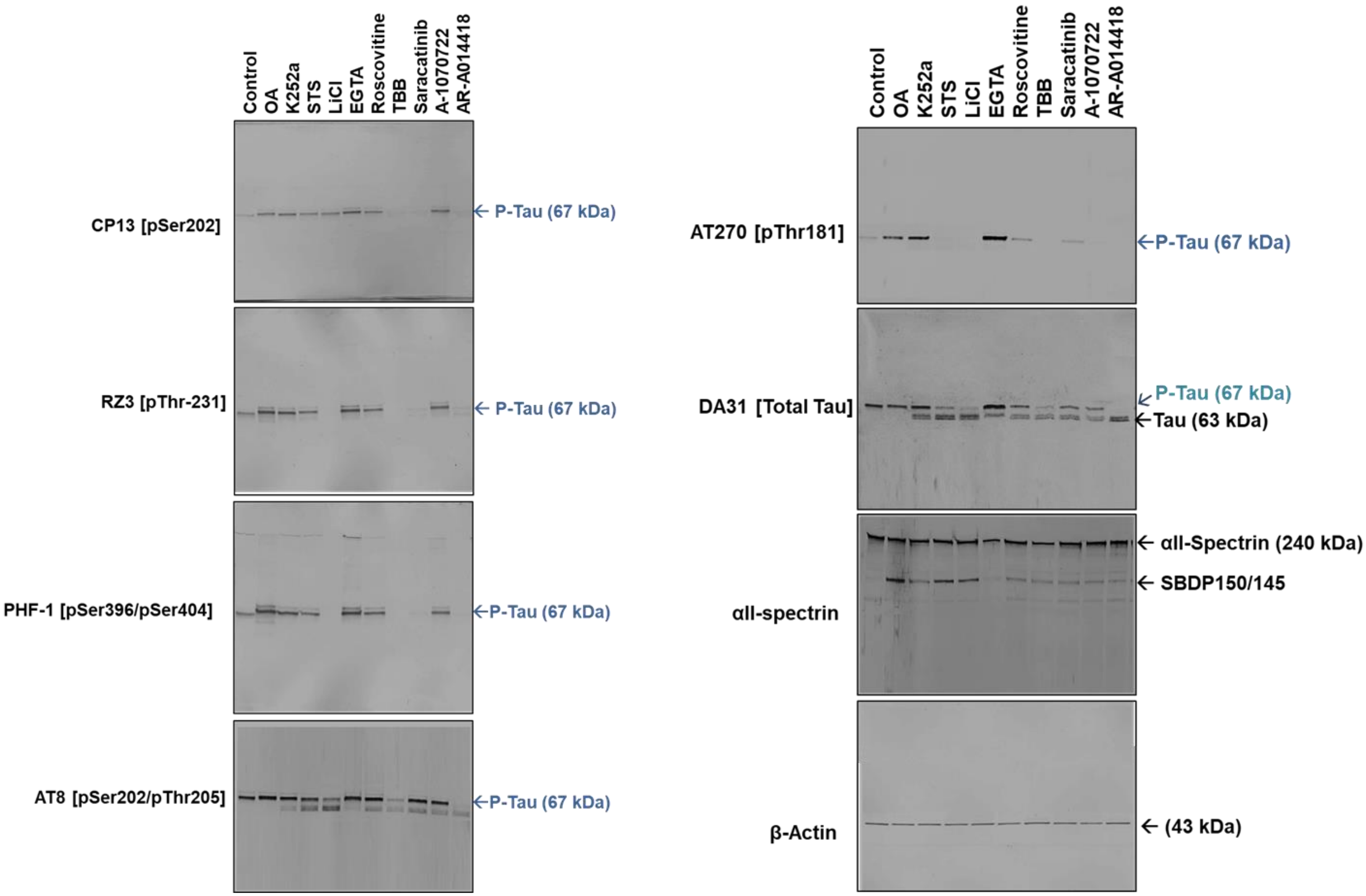

Raw image for the production of Fig 5a. Images were captured with the computer-assisted densitometric *scanning* (*Epson* 8836XL high-resolution *scanner* and NIH Image J densitometry software. MW, GE Healthcare rainbow full range molecular weight marker.
